# Supplementary material for: The impact of withdrawing aquaculture facilities on metazooplankton communities in the lakes are connected to the Yangtze River, China
Source: PLoS One. 2021 May 26;16(5):e0252100. doi: 10.1371/journal.pone.0252100 (PMC8153418; doi:10.1371/journal.pone.0252100)

PLOS ONE

The effect of the removal of enclosure aquaculture on metazooplankton communities in the lakes connected to the Yangtze River in China

--Manuscript Draft--

**Manuscript Number:**	PONE-D-21-05633
**Article Type:**	Research Article
**Full Title:**	The effect of the removal of enclosure aquaculture on metazooplankton communities in the lakes connected to the Yangtze River in China
**Short Title:**	no,thanks.
**Corresponding Author:**	Zhongze Zhou<br>Anhui University<br>Hefei, CHINA
**Keywords:**	Metazoan zooplankton (Metazooplankton); Aquatic plants; Diversity; Enclosure aquaculture; Huayanghe Lakes; The Yangtze River
**Abstract:**	In order to clarify the response mechanism of metazooplankton to the improvement of the water environment after the removal of purse seine, we studied the dynamic changes of the community structure of metazooplankton in the lakes connected to the Yangtze River after the removal of enclosure aquaculture. During our study of the Huayanghe Lakes, the highest regional (γ) diversity was 71 in summer, while the lowest was 32 in winter. Species turnover in space (β diversity) varied between 10.01 and 56.52, which was highest in summer. We clearly saw temporal and spatial variations in the metazooplankton community with the highest mean cladocera density observed in spring, at 56.67 ind./L. The density gradually decreased to the lowest in winter. The mean copepoda density started at 12.94 ind./L in spring and swelled to 24.11 ind./L in autumn. The mean rotifera density fluctuated from 170.54 to 2723.20 ind./L, which accounted for an absolute proportion of the total density of metazooplankton in each sampling season. Redundancy analysis revealed that environmental factors, such as transparency, Chl α, water temperature and water depth, had significantly influenced the metazooplankton community structure.
**Order of Authors:**	Yixing Zhang
	Yutao Wang
	Zhongze Zhou
**Additional Information:**	
**Question**	**Response**
**Financial Disclosure**<br><br>Enter a financial disclosure statement that describes the sources of funding for the work included in this submission. Review the submission guidelines for detailed requirements. View published research articles from *PLOS ONE* for specific examples.<br><br>This statement is required for submission and **will appear in the published article** if the submission is accepted. Please make sure it is accurate.	This work was supported by the Joint Research Project for the Yangtze River Conservation (Phase I), China (No.2019-LHYJ-01-0212, 2019-LHYJ-01-0212-17).

Powered by Editorial Manager® and ProduXion Manager® from Aries Systems Corporation

**Unfunded studies**<br>Enter: *The author(s) received no specific funding for this work.*<br><br>**Funded studies**<br>Enter a statement with the following details:<br>• Initials of the authors who received each award<br>• Grant numbers awarded to each author<br>• The full name of each funder<br>• URL of each funder website<br>• Did the sponsors or funders play any role in the study design, data collection and analysis, decision to publish, or preparation of the manuscript?<br>• **NO** - Include this sentence at the end of your statement: *The funders had no role in study design, data collection and analysis, decision to publish, or preparation of the manuscript.*<br>• **YES** - Specify the role(s) played.<br><br>* typeset	
**Competing Interests**<br><br>Use the instructions below to enter a competing interest statement for this submission. On behalf of all authors, disclose any competing interests that could be perceived to bias this work—acknowledging all financial support and any other relevant financial or non-financial competing interests.<br><br>This statement is required for submission and **will appear in the published article** if the submission is accepted. Please make sure it is accurate and that any funding sources listed in your Funding Information later in the submission form are also declared in your Financial Disclosure statement.<br><br>View published research articles from *PLOS ONE* for specific examples.	NO authors have competing interests.

**NO authors have competing interests**<br><br>Enter: *The authors have declared that no competing interests exist.*<br><br>**Authors with competing interests**<br><br>Enter competing interest details beginning with this statement:<br><br>*I have read the journal's policy and the authors of this manuscript have the following competing interests: [insert competing interests here]*<br><br>* typeset	
**Ethics Statement**<br><br>Enter an ethics statement for this submission. This statement is required if the study involved:<br><br>• Human participants<br>• Human specimens or tissue<br>• Vertebrate animals or cephalopods<br>• Vertebrate embryos or tissues<br>• Field research<br><br>Write "N/A" if the submission does not require an ethics statement.<br><br>General guidance is provided below. Consult the submission guidelines for detailed instructions. **Make sure that all information entered here is included in the Methods section of the manuscript.**	N/A

**Format for specific study types**<br><br>**Human Subject Research (involving human participants and/or tissue)**<br>• Give the name of the institutional review board or ethics committee that approved the study<br>• Include the approval number and/or a statement indicating approval of this research<br>• Indicate the form of consent obtained (written/oral) or the reason that consent was not obtained (e.g. the data were analyzed anonymously)<br><br>**Animal Research (involving vertebrate animals, embryos or tissues)**<br>• Provide the name of the Institutional Animal Care and Use Committee (IACUC) or other relevant ethics board that reviewed the study protocol, and indicate whether they approved this research or granted a formal waiver of ethical approval<br>• Include an approval number if one was obtained<br>• If the study involved *non-human primates*, add *additional details* about animal welfare and steps taken to ameliorate suffering<br>• If anesthesia, euthanasia, or any kind of animal sacrifice is part of the study, include briefly which substances and/or methods were applied<br><br>**Field Research**<br><br>Include the following details if this study involves the collection of plant, animal, or other materials from a natural setting:<br>• Field permit number<br>• Name of the institution or relevant body that granted permission	
**Data Availability**<br><br>Authors are required to make all data underlying the findings described fully available, without restriction, and from the time of publication. PLOS allows rare exceptions to address legal and ethical concerns. See the PLOS Data Policy and FAQ for detailed information.	Yes - all data are fully available without restriction

A Data Availability Statement describing where the data can be found is required at submission. Your answers to this question constitute the Data Availability Statement and **will be published in the article**, if accepted.<br><br>**Important:** Stating 'data available on request from the author' is not sufficient. If your data are only available upon request, select 'No' for the first question and explain your exceptional situation in the text box.<br><br>Do the authors confirm that all data underlying the findings described in their manuscript are fully available without restriction?	
**Describe where the data may be found in full sentences. If you are copying our sample text, replace any instances of XXX with the appropriate details.**<br><br>• If the data are **held or will be held in a public repository**, include URLs, accession numbers or DOIs. If this information will only be available after acceptance, indicate this by ticking the box below. For example: *All XXX files are available from the XXX database (accession number(s) XXX, XXX.*).<br>• If the data are all contained **within the manuscript and/or Supporting Information files**, enter the following: *All relevant data are within the manuscript and its Supporting Information files.*<br>• If neither of these applies but you are able to provide **details of access elsewhere**, with or without limitations, please do so. For example:<br><br>*Data cannot be shared publicly because of [XXX]. Data are available from the XXX Institutional Data Access / Ethics Committee (contact via XXX) for researchers who meet the criteria for access to confidential data.*<br><br>*The data underlying the results presented in the study are available from (include the name of the third party*	Data cannot be shared publicly because of it is used for other writing.

and contact information or URL).<br>• This text is appropriate if the data are owned by a third party and authors do not have permission to share the data.<br><br>* typeset	
Additional data availability information:	

Powered by Editorial Manager® and ProduXion Manager® from Aries Systems Corporation

Manuscript Click here to access/download;Manuscript;Paper.docx

The effect of the removal of enclosure aquaculture on metazooplankton communities in the lakes connected to the Yangtze River in China

Yixing Zhang, Yutao Wang, Zhongze Zhou*

School of Resources and Environmental Engineering, Anhui University, Hefei 230601, PR China

* Corresponding author at: School of Resources and Environmental Engineering, Anhui University, Hefei 230601, PR China.

E-mail address: zhzz@ahu.edu.cn.

Abstract:

In order to clarify the response mechanism of metazooplankton to the improvement of the water environment after the removal of purse seine, we studied the dynamic changes of the community structure of metazooplankton in the lakes connected to the Yangtze River after the removal of enclosure aquaculture. During our study of the Huayanghe Lakes, the highest regional (γ) diversity was 71 in summer, while the lowest was 32 in winter. Species turnover in space (β diversity) varied between 10.01 and 56.52, which was highest in summer. We clearly saw temporal and spatial variations in the metazooplankton community with the highest mean cladocera density observed in spring, at 56.67 ind./L. The density gradually decreased to the lowest in winter. The mean copepoda density started at 12.94 ind./L in spring and swelled to 24.11 ind./L in autumn. The mean rotifera density fluctuated from 170.54 to 2723.20 ind./L, which accounted for an absolute proportion of the total density of metazooplankton in each sampling season. Redundancy analysis revealed that

environmental factors, such as transparency, Chl α, water temperature and water depth, had significantly influenced the metazooplankton community structure.

Keywords: Metazoan zooplankton (Metazooplankton); Aquatic plants; Diversity; Enclosure aquaculture; Huayanghe Lakes; The Yangtze River

1. Introduction

The Anhui section of the Yangtze River basin is located in the mid-subtropical zone, and there are large areas of shallow lakes on both sides. These large shallow lakes along the Yangtze River in Anhui province are important places for fish migration and breeding along the east coast of China, habitats for winter migratory birds, and important gathering places for migratory birds from East Asia to Australia (Xu et al., 2008). Since 2000, due to the rapid development of aquaculture industry, the aquatic plants of the lake have been seriously damaged by enclosure aquaculture, especially the submerged plants which have almost disappeared, and the habitat of waterbirds has been seriously degraded, seriously affecting the safety of the entire lake wetland ecosystem (Zhu et al., 2010).

Metazooplankton, mainly composed of rotifera, cladocera and copepoda, feed mainly on smaller phytoplankton, protozoa, bacteria and some organic detritus, and are important primary consumers in aquatic ecosystems. Metazooplankton is also an economic aquatic animal, which can be used as important food for fish and other large aquatic organisms. Therefore, metazooplankton play an important role in energy flow and material circulation in aquatic ecosystems (Zhang, 2019). Compared with other aquatic animals, they are small but numerous, sensitive to the living environment and have strong metabolic activity

(Špoljar et al., 2016). And the change of their community structure can effectively reflect the nutrient status of the water body (Liu et al., 2018; Jernberg et al., 2017). Changes in metazooplankton community structure may be influenced by many environmental factors in the water environment, including abiotic conditions such as water temperature, water level, light, dissolved oxygen content, aquatic vascular plants in the same habitat, emergent, floating, floating-leaved, and submerged plants, as well as other biological conditions such as phytoplankton, higher aquatic animal (fish, shrimp, etc.) and benthic organisms (Wang et al., 2018; Zeng et al., 2018; Goździejewska et al., 2016; Lin et al., 2014 and Zhao et al., 2013).

Huayanghe Lakes in Anhui play an important ecological function in the Yangtze River basin. However, from 2007 to 2017, the rapid development of a fishery was gradually destroying the submerged plants. Since 2018, with the strong support of the national great protection policy for the Yangtze River, the local government removed the aquaculture seine in the Huayanghe Lakes, and the aquatic plants were quickly restored, and the number of birds populations increased. Fishes that were cultivated by high-density economic fish in the form of artificial seine converted to natural reproduction, and the density was greatly reduced. Field survey results find that the rapid restoration of aquatic plants in Lake Huangda in summer is dominated by the floating-leaved plants *Trapa incisa*, whose coverage can reach more than 95%, and the distribution area of aquatic plants such as *Zizania caduciflora* and *Nelumbo nucifera* by the lake can reach 90%. According to the data measured by Zhang et al. (2019), secchi depth (SD), total nitrogen (TN), and dissolved oxygen (DO) content of Lake Huangda were 0.37 m, 1.46 mg/L, and 9.40 mg/L

respectively; in the 2018 survey, they were 0.48 m, 0.73 mg/L, and 10.00 mg/ L, indicating that the water quality improved after the removal of the seine. Susong County's water production dropped sharply from 90400 t in 2017 (http://www.susongbbs.com/1408365-1-1.html) to 83900 t in 2018 (http://www.ahmhxc.com/tongjigongbao/14909_2.html). Based upon the data cited above, further studies should be conducted to clarify the impact of the removal of the fishery purse seine on the metazooplankton in the lakes connected to the Yangtze River.

From summer 2018 to spring 2019, we monitored the restoration of aquatic plants, parameters of lake environmental factors, composition, density and species diversity of metazooplankton with the purpose of: (1) analyzing the impact of aquatic plants recovering rapidly after the removal of enclosure aquaculture on water environment and meatzooplankton; and (2) evaluating the main ecological factors influencing metazooplankton. The study is expected to provide a scientific basis for the restoration and management of lake water ecosystems in the Yangtze River basin.

2. Material and method

2.1 Description of study area

Huayanghe Lakes is a natural lake, and its plants distribution pattern was gradually distributed from emergent plants and floating-leaved plants at the lake edge to submerged plants in the lake area. From 2000 to the end of 2017, enclosure aquaculture seriously degraded the aquatic plants of the Huayanghe Lakes.

According to the requirements of the biological water environment monitoring part of "The Environmental Monitoring Technical Specifications" (Zhang and Huang, 1991) and based on the size and shape characteristics of each lake in the Huayanghe Lakes, we set up a total of 26 sample points (Fig 1, including seven sites in Lake Bo, nine sites in Lake Huangda and ten sites in Lake Longgan) in the lakes and collected samples once per season from August 2018 to April 2019.

2.2 Sampling collection and treatment

Metazooplankton is mainly composed of rotifera, cladocera and copepoda. We collected the qualitative samples of rotifera by the net towing method (No.25 plankton net with a pore size of 64 μm), and 1L of mixed water samples for the quantitative samples. On site, we added Lugol iodine solution for fixation. We brought the samples back to the laboratory for standing precipitation for 48 h, concentrated to 30 mL and counted them under a microscope.

The collection method of qualitative samples of cladocera and copepoda is the same as that of rotifera qualitative samples. We collect the quantitative samples by filtering at least 10 L of mixed water through a 64 μm mesh, and then concentrate to 50 mL plastic bottles. A 4% formaldehyde solution is added immediately on the scene. Using a microscope in the laboratory, we identify and count the species. The counting method of metazooplankton mainly refers to "Freshwater Plankton Research Method" for counting. The species identification mainly refers to the description of metazooplankton by Wang (1961), Jiang and Du (1979), and Shen (1979).

At each sampling site, we collected an appropriate amount of water samples while taking metazooplankton samples. We stored the water samples at low temperature and brought them back to the laboratory according to the Surface Water Environmental Quality Standards (GB 3828-2002) for the measure of total nitrogen (TN), total phosphorus (TP), ammonium nitrogen (AN), nitrate nitrogen (NN) and other indicators. Using the 90% acetone extract+spectrophotometry measurement, we analyzed the Chlorophyll.α (Chl α) concentration, and then measured the turbidity (Turb) with the turbidimeter. We conducted an on-site measurement of water depth (WD), water temperature (WT), transparency (SD), dissolve oxygen (DO), conductivity (Cond) and other water quality parameters.

2.3 Data analyses

In order to describe and explain the difference of the metazooplankton communities among each seasonal sample, biodiversity indices are computed according to Dube et al. (2017). Another name for γ diversity is regional diversity. This measures the total number of species encountered at each sampling occasion (Chaparro et al., 2015). The second diversity index is α diversity (α). This determines the average number of species under each local habitat (Simoes et al., 2013; Chisholm et al., 2011), also known as local diversity. The last index is β diversity, which represents the species turnover. The value is calculated according to the modified formula of Harrison et al. (1992). The formula is: $\beta = \{[(\gamma/\alpha_{ave})-1]/(N-1)\}*100$, where N is the number of lakes in each sampling period and α_{ave} is the mean α diversity. It varies between 0 (complete similarity: all regional species occur in all habitats) and 100 (complete dissimilarity: each species occurring in a single habitat).

The dominant species of metazooplankton, determined based on the dominance value of each species, was computed by $Y=(n_i/N)*f_i$, where n_i is the abundance of individual species i, N is the total number of all species, and f_i is the occurrence frequency of species i. When $Y \geq 0.02$, this species is the dominant species (Xu et al., 1995).

We plotted the diagrams using Microsoft Excel and Origin and performed a Pearson correlation analysis by applying the SPSS package (Version 20.0, IBM http://www.ibm.com) to evaluate the influence of the limnological variables on metazooplankton densities. To analyze the relationship between the metazooplankton community and the environmental factors, we used CANOCO software. With the ArcGIS software (Version 10.0, ESRI http://www.esri.com), we drew the vegetation distribution map and constructed the interpolation map by the kriging method.

3. Results

3.1 Ecological factors parameters

Tab. 1 shows our examination of the physicochemical parameters of the Huayanghe Lakes. There were obvious differences in water temperature in the four seasons, the highest in summer and the lowest in winter, with a range of 4.5~34℃. The water was slightly alkaline on the whole with a pH value of 8.50±0.43, and there was no significant difference between seasons ($p>0.05$). The average conductivity was 138.11~184.87 us/cm. In one year, transparency generally increased appreciably with increasing water depth ($p<0.05$), and turbidity and transparency showed a significant negative correlation ($p<0.01$).

Monitoring results exhibited that (Tab 1), the TP and TN concentrations in each season were considerably different ($p<0.05$). The concentrations of TN and TP in Lake Huangda was the lowest in summer, at 0.48 mg/L and 0.005 mg/L respectively; the maximum Chl α concentration in Lake Longgan was 9.91 ug/L. The ratio of TN/TP (19.48±18.99) experienced a notable change, and the change of nutrition inevitably affected the growth of phytoplankton, thus affecting the zooplankton community structure. The concentration of Chl α was highest in summer (5.09 ug/L) and lowest in winter (2.18 ug/L).

Tab 1. Seasonal variation of physical and chemical factors (mean±SD) in the Huayanghe Lakes.

Parameters	2019.04/Spring	2018.08/Summer	2018.11/Autumn	2019.01/Winter
WT/°C	25.03±1.48	30.80±1.23	15.10±0.90	5.31±0.39
pH	8.78±0.34	8.25±0.42	8.57±0.36	8.38±0.31
DO/(mg/L)	9.46±1.12	8.68±0.77	11.09±0.68	13.24±0.33
Cond/(us/cm)	184.87±27.24	138.11±24.15	156.71±21.38	162.46±24.53
SD/cm	19.65±17.50	59.28±40.66	57.30±22.11	27.54±22.43
DW/m	1.45±0.41	2.98±0.48	2.30±0.54	2.15±0.49
Turb/NTU	250.17±262.04	28.35±21.56	25.19±12.39	197.32±121.49
Chl α/(ug/L)	3.26±2.52	5.09±5.19	4.28±4.12	2.18±1.69

AN/(mg/L)	0.48±0.41	0.08±0.04	0.47±0.16	0.20±0.10
NN/(mg/L)	0.18±0.19	0.04±0.03	0.05±0.02	0.13±0.05
TN/(mg/L)	0.94±0.24	0.56±0.32	1.06±0.39	0.73±0.22
TP/(mg/L)	0.18±0.10	0.01±0.01	0.10±0.05	0.12±0.04

In 2015 and 2016, the aquatic plants monitoring results for two years noted that in summer, Ass. N. *nucifera* and Ass. Z. *caduciflora* were a zonal distribution in the coastal waters of Lake Bo; Ass. T. *incisa* was mainly distributed in the north and south of Lake Huangda in the east of Lake Longgan and the north of Lake Bo. Ass. *Potamogeton Wrightii* was distributed sporadically in the middle of Lake Huangda, and the aquatic plants distribution area was not more than 20% (Fig 2a).

At the end of 2017, the aquaculture seine of the Huayanghe Lakes was removed, and the aquatic plants quickly recovered. During the investigation in the summer of 2018, it was found that the coverage of aquatic plants in each lake increased significantly. The dominant species in the coastal waters were emergent plants such as N. *nucifera* and Z. *caduciflora*, and the accompanying species were floating plants such as *Hydrocharis dubia* and *Salvinia natans*. However, the distribution of the aquatic plants in each lake area was appreciably different. There were surveyed that the submerged plants in the area near Lake Bo were mainly *Vallisneria natans*, *Ceratophyllum demersum* and *Myriophyllum verticillatum*. There were small areas of T. *incisa* and floating *Eichhornia crassipes* in Lake Longgan in the boundary waters of Hubei. In Lake Huangda, however, the floating-leaved plants T.

incisa and *Trapa bispinosa* germinated rapidly and became dominant species, and their distribution area was 20970 hm^2 in Lake Huangda, accounting for more than 87% of the total area of the lake (Fig 2b).

3.2 Metazooplankton diversity indices and dominant species

The highest γ diversity was recorded in summer, with a total of 71 zooplankton taxa: 45 Rotifera, 17 Cladocera, and 9 Copepoda (Fig 3). The lowest γ diversity was 32, including 18 Rotifera, 7 Cladocera, and 7 Copepoda in winter. In addition, the γ diversity in spring (51 taxa: 23 Rotifera, 18 Cladocera, and 10 Copepoda) was higher compared to that in autumn (45 taxa: 25 Rotifea, 12 Cladocera, and 8 Copepoda). Similarly, there was a significant trend for the highest α diversity (52.67±7.53) to be in summer than during the other sampling seasons. The lowest α diversity was 18.00±3.95 in winter. Species turnover (β diversity) in the space exhibited a trend of first increasing and then decreasing, which was spring (26.51), summer (56.51), autumn (14.51) and winter (10.01).

The development of the metazooplankton community and the dominant species groups showed hard variation in each studied season. But, Nauplii was always present in the three lakes on all sampling occasions. And most of the dominant rotifera were the indicator species of the middle fouling zone, such as *Branchionus budapestiensis*, *Keratella cochlearis*, *Brachionus forficula*, etc. We recorded the same dominant groups, including *Trichocerca pusilla*, K. *cochlearis*, P. *trigla* and *Bosmina longirostris*, in the three lakes during the sampling period of the summer of 2018. *Monostyla lunaris*, found only in Lake Longgan, contributed the most among the dominant species. B. *budapestiensis* and

Ascomorpha ecaudis contributed more to the metazooplankton community in spring 2019, and *Daphnia cucullata* was recorded in samples. Throughout the lakes survey in the autumn of 2018, we observed *Keratella quadrata*, *Ascomorpha saltans*, A. *ecaudis*, and *Microcyclops vaticans*. In addition, K. *quadrata*, P. *trigla*, B. *angularis*, *Branchionus clycifolrus* and *Asplachna priodonta* dominated the metazooplankton groups in the studied lakes in winter 2018.

3.3 Temporal and spatial variation in metazooplankton community

Rotifera always dominated the metazooplankton community during the sampling phases. The density accounted for more than 80% at each season (Fig 4). The relative density of cladocera was highest in spring (2.65%), while that of copepoda was highest in autumn (11.91%). We found the higher mean cladocera density (56.67 ind./L and 25.36 ind./L) in spring and summer, and watched it gradually decrease to its lowest in winter. The mean copepoda density ranged from 12.94 ind./L in spring to 24.11 ind./L in autumn. The total rotifera density was significantly higher than that of the other two crustacean zooplankton ($p<0.01$), within the range of 170.54-2723.20 ind./L.

It is shown in Fig 5 that there was a significant spatial difference in the annual average density of metazooplankton in different lakes. The level of metazooplankton in Lake Longgan was generally on the high value and gradually declined along a certain gradient; the highest annual average density was 4361.0 ind./L. While the overall level of Lake Bo was low, the lowest value was 294.6 ind./L.

3.4 Relationship between metazooplankton and environmental factors

Using Canoco, we first performed a detrended correspondence analysis (DCA) between the densities of 17 major metazooplankton species and environmental factors, and the results showed that the length of the maximum gradient in the sequencing axis was 2.1 (<3), which means that there was a linear relationship between metazooplankton and environmental factors. So redundant analysis (RDA) was used. After the Monte Carlo replacement detection, we performed an RDA between main species and environmental factors.

RDA results exhibited that the eigenvalues of axis 1 and axis 2 were 0.4152 and 0.0836, respectively. The linear combinations of environmental factors making up the first two RDA axes explained 31.9% of the metazooplankton assemblage data variance. It can be seen from Fig 6 that transparency (38.6%), water depth (12.9%), and Chl α (11.1%) are closely related to the community structure and annual average density of metazooplankton. M. *lunaris*, B. *forficula*, and the mean annual density of metazooplankton were positively correlated with Chl α, TN, and Cond, while T. *hyalinus* was negatively correlated with them, which may be difficult to sustain the mass reproduction of phytoplankton. WT was the main influencing factor for K. *cochlearis* and K. *quadrata*. A. *saltans*, A. *ecaudis*, and T. *hyalinus* were positively correlated with TP and pH, and a negative association with SD, and may prefer to live in water with high degree of nutrition. Therefore, TN, TP and Cond were also important factors affecting the metazooplankton community in the Huayanghe Lakes.

4. Discussion

4.1 Effect of the removal of enclosure aquaculture on aquatic plants and water environment

Through the investigation after the removal of enclosure aquaculture in Huayanghe Lakes, the aquatic plants restoration of each sub-lake was obviously different. Submerged plants were the main species in Lake Bo, and floating-leaved plants were the main species in Lake Huangda, while aquatic plants in Lake Longgan were fewer. This was different from before enclosure aquaculture (Fig 2). The plants were mainly distributed along the lakeshore and the river mouth, and the submerged plants basically disappeared in the open water area of each lake. On the one hand, the reason for this may be the reduction of fish density, especially the reduction of grass-feeding economic fish mainly composed of *Ctenopharyngodon idella*, *Parabramis pekinensis*, and *Megalobrama amblycephala*, which reduces the destructive effect on fruits and seedlings of aquatic plants and increased the possibility of their germination and growth (Zhong et al., 2001). Yang and Li (1996) reported in the high-density seine fish culture test area in Lake East Taihu that aquatic plants were degraded due to fish culture pollution. On the other hand, habitat connectivity can maintain the integrity of wetland ecosystem and encourage the diversity of aquatic organisms. Aquatic plants transform water and inorganic nutrients (mainly nitrogen and phosphorus) into organic matter through photosynthesis, while becoming food for consumers themselves. Aquatic plants and phytoplankton together constitute the primary producer in the lake ecosystem, which are the starting point of the food chain and play an important role in the material cycle and energy flow. Therefore, aquatic plants have an important influence on the structure and function of the lake ecosystem.

Analyzing the results of the water quality survey after the removal of purse seine in the Huayanghe Lakes, it was found that, compared with the previously published data by Xu (2016) and Zhang et al. (2019), the TN and TP contents were significantly reduced in summer, but the overall trend was increasing. Xue et al. (2010) found that, after the removal of the aquaculture net in Lake Dianshan, the concentration of TN and TP in Kunshan water increased in general, SD decreased, and DO also increased to a certain extent. It might be due to the presence of large aquatic plants, which absorbs and assimilates nutrients such as carbon, nitrogen and phosphorus during its growth (Xu et al., 2005), fixed sediments and mud, and reduces wind and wave action (Meyer et al., 2019). However, in the process of seasonal decay, large aquatic plants will also release their own nutrients into the water body, leading to the increase of nutrients. Kosten et al. (2009) demonstrated that the presence of aquatic plants can lead to significant small-scale reductions in nutrients, Chl α and Trub in or above the plants layer compared to sites outside the plants.

Chl α represents phytoplankton biomass, and the highest Chl α was detected in Lake Longgan. The field investigation observed that there was almost no large submerged plants in Lake Longgan, which was a mainly shallow lake dominated by phytoplankton with turbidity state, and the water quality was relatively poor. As phytoplankton covered almost the whole lake, light could not enter the water, and aquatic plants seedlings were unable to photosynthesis. This severely inhibits the growth of aquatic plants. Huo et al. (2010) carried out ecological restoration in Lake Dishui, Shanghai, using *Daphnia magna* to control algal propagation and increase water transparency, and successfully cultivated large

aquatic plants. We observed small areas of T. *incisa* and scattered floating E. *crassipes* in the Hubei boundary of Longgan Lake. The study by Jiang et al. (2008) mentioned that floating plants had low environmental requirements and pollution-resistant, and can grow in an environment with high nitrogen and phosphorus levels, especially some fast growing floating plants, such as E. *crassipes* and *Lemna minor*. They are widely used to treat eutrophication water bodies to reduce nitrogen and phosphorus levels, improve water transparency, and gradually restore other aquatic plants and the whole ecological environment.

4.2 Effects of aquatic plants restoration on metazooplankton after the removal of enclosure aquaculture

The distribution of metazooplankton communities depends to a large extent on the micro-environmental characteristics of water bodies, and large aquatic plants can provide micro-environments (such as space, food resources, etc.) for metazooplankton (Choi et al., 2014). In addition to their ability to rapidly absorb nutrients from water and sediment, aquatic plants can alter some of the physical and chemical environmental factors that affect the distribution of metazooplankton, such as transparency, pH, disturbance, etc.; it can affect phytoplankton, which are important food sources for metazooplankton (Zheng and Sugie, 2019); it also provides shelter for metazooplankton (Meyer et al., 2019), thus affecting fish feeding (Bolduc et al., 2016). Therefore, aquatic plants can affect the habitats of metazooplankton due to many aspects, and are one of the important factors that affect the species composition, density and diversity of metazooplankton (Chen et al., 2007a). A total of 82 species of metazooplankton were identified in this study, including 49 species of

rotifera, 33 species of cladocera and copepoda, with an average density of 202.39-2786.70 ind./L. According to the data measured by Xu (2016) from April 2015 to January 2016, a total of 53 species of metazooplankton were discovered in the same lake, including 39 species of rotifera, with an average density of 223.08-2061.62 ind./L. During the two years from April 2015 to January 2017, Zhang et al. (2019) discovered 25 species of crustacean zooplankton. This indicated that, on the whole, the species diversity and density of metazooplankton were on the rise. The number of large crustaceans also increased significantly, but rotifera were still the dominant species. This may be because that, in the early stage of submerged plants recovery, planktonic algae are still the main primary producers, and that the secondary producers depending on phytoplankton still exist in large numbers (Ghadouani et al., 2003 and Bergquist et al., 1985). According to some researchers (Miao et al., 2013 and Chen et al., 2008), the species abundance and diversity of metazooplankton were generally higher in waters with aquatic plants coverage than in those without plants. Hu's (1999) research on metazooplankton in five lakes, including Baoan Lake in Hubei in the middle and lower reaches of the Yangtze River, discovered that metazooplankton species and density were closely related to aquatic vegetation.

In our study, the average annual density of metazooplankton had a gradient variation (Fig 5) with a high β diversity (>10), indicating that there were significant spatial differences in the metazooplankton community; especially in summer, species richness of crustacean zooplankton were higher in Lake Bo (26 species) and Lake Huangda (21 species) than in Lake Longgan (18 species). Lake Longgan had the lowest vegetation coverage among the Huayanghe Lakes, and the submerged plants had a better vegetation recovery in

Lake Bo, while the floating-leaved plants coverage was the highest in Lake Huangda. Zeng Lei et al. (2018) reported in their study that species richness of large zooplankton were obviously enhanced in the restoration process of aquatic plants. It has been also found that the proportion of metazooplankton was highest in cladocera and copepoda in Lake Bo. Compared with Xu (2016), *Sida erystallina* and *Bosminopsis deitersi* were widely distributed, and species number of cladocera increased (Daphnia from the original only D. *cucullata* to the presence of *Daphnia magna*, *Daphnia obtusa*, *Daphnia hyalina* and *Daphnia pulex*). This may be due to the existence of large aquatic plants and the increase of coverage, coupled with the obvious increase of water transparency in Lake Huangda and Lake Bo. It has been uncovered that large-size species like to grow in aquatic plants, such as S. *erystallina* and B. *deitersi*, which appear in demonstration areas of aquatic plants restoration (Chen et al. 2007b). Zhang et al. (2018) proposed that the change of aquatic plants coverage was one of the important factors affecting the species composition of the cladocera community, and that lush aquatic plants could provide shelters for cladocera. After the removal of enclosure aquaculture, the metazooplankton communities were still dominated by the indicator species of the middle fouling zone, such as B. *budapestiensis*, B. *forficula* and K. *cochlearis* (Chen et al., 2011). However, compared with Zhang et al. (2019) who recorded that γ_{max} was 52 (including Lake Wuchang) in May 2016, the γ_{max} diversity (71) in this study has markedly increased.

4.3 Effects of fish on metazooplankton after the removal of enclosure aquaculture

The fish is located at the top of the food chain of the aquatic ecosystem. This has an important influence on the structure change of metazooplankton community. The research

of Yang et al. (2014) proved that fish preyed on metazooplankton selectively and generally gave preference to larger individuals under the same conditions. Susong County's water production was 90400 t in 2017, compared with 83900 t in 2018. After the removal of the purse seine, the output of fish decreased obviously, reducing the predation pressure on the metazooplankton. Both the abundance of metazooplankton and the species of cladocera and copepoda increased markedly. New species, such as D. *magna*, D. *hyalina*, S. *erystallina*, *Cyclops vicinus* and *Microcyclops intermedius*, were observed. Fish stocking density also plays an important role in zooplankton community structure (Jiang et al., 2013; Nicolle et al., 2011).

4.4 Effect of water environment improvement on metazooplankton after the removal of enclosure aquaculture

Pearson correlation analysis and RDA displayed that the main physical and chemical factors affecting the structure of the metazooplankton community in the Huayanghe Lakes after the removal of purse seine were SD, water temperature, Chl α, electrical conductivity, etc., which was consistent with many other research results (Lin et al., 2018; Zeng et al., 2016).

The restoration of aquatic plants led directly or indirectly to the changes of physicochemical factors in the Huayanghe Lakes, which led to the transformation of the structure of the metazooplankton community. WT was an important environmental factor affecting the growth, development, community composition, and population distribution of metazooplankton (Morse et al., 2016; Roemmich and McGowan, 1995). During this study,

it was found that the number of metazooplankton species was most affected by the species of rotifera, and the density of rotifera accounted for more than 80% at each season (Fig 4). The variation trend of the two was basically the same, which was the same as the research results of other periods of the Huayanghe Lakes (Zhang et al., 2019; Xu, 2016). This research area had a humid subtropical climate with obvious monsoon and low water temperature in autumn and winter. These conditions are not conducive to the growth and reproduction of metazooplankton, and the species and density are relatively few. However, with the increase of water temperature, resting eggs continued to hatch, Nauplius appeared in large numbers, cladocera and copepoda species increased. Lin et al. (2014) uncovered that metazooplankton was unstable at different sampling cycles in Lake Dishui. From winter to summer, with the increase of temperature and the decrease of turbidity, the species of metazooplankton increased. Meanwhile, their density increased as well. From summer (WT=30.80℃) to autumn (WT=15.10℃), there was a significant decrease in the density and γ diversity of metazooplankton. This may be due to seasonal changes in summer and autumn and large changes in water temperature. Through RDA and Pearson correlation analysis, we turned up that transparency and water depth had significant effects on the density of the major species. Hart's (2010) study demonstrated that metazooplankton was closely related to transparency and turbidity. The low transparency could inhibit the development of metazooplankton. Some researchers (Wang et al., 2016; Huo et al., 2010) had discovered that the increase in metazooplankton grazing can help improve transparency.

In this study, Chl α concentration was also one of the important influencing factors, and

RDA diagram showed that Chl α concentration, TN and TP were pronouncedly correlated with most metazooplankton species and the annual average density. Phytoplankton is one of the important food sources of zooplankton, and nutrients such as nitrogen and phosphorus affect metazooplankton mainly by affecting phytoplankton.

Acknowledgements

This work was supported by the Joint Research Project for the Yangtze River Conservation (Phase Ⅰ), China (No.2019-LHYJ-01-0212, 2019-LHYJ-01-0212-17). We thank Kun Zhang, An Li and Yanling Guo for their participation in the field work.

References

Bolduc, P., Bertolo, A., Pinel-Alloul, B., 2016. Does submerged aquatic vegetation shape zooplankton community structure and functional diversity? A test with a shallow fluvial lake system. Hydrobiologia 778(1), 151-165. https://doi.org/10.1007/s10750-016-2663-4.

Bergquist, A.M., Carpenter, S.R., Latino, J.C., 1985. Shift in phytoplankton size structure and community composition during grazing by contrasting zooplankton assemblages. Limnol. Oceanogr. 30, 1037-1045. https://doi.org/10.4319/lo.1985.30.5.1037.

Chaparro, G., Kandus, P.,O., Farrell, I., 2015. Effect of spatial heterogeneity on zooplankton diversity: a multi-scale habitat approximation in a floodplain lake. River Res. Appl. 31, 85-97. https://doi.org/10.1002/rra.2711.

Chisholm, C., Lindo, Z., Gonzalez, A., 2011. Metacommunity diversity depends on connectivity and patch arrangement in heterogeneous habitat networks. Ecography 34, 415-424. https://doi.org/10.1111/j.1600-0587.2010.06588.x.

Choi, J.Y., Jeong, K.S., Kim, S.K., La, G.H., Chang, K.H., Joo, G.J., 2014. Role of macrophytes as microhabitats for zooplankton community in lentic freshwater ecosystems of South Korea. Ecol. Inform. 24,177-185. https://doi.org/10.1016/j.ecoinf.2014.09.002.

Chen, G.R., Liu, Z.W., Zhong, P., Li, J.H., Yan, X.W., Liu, P.P., 2007a. The situation and relation of mycrophyte, zooplankton and fish in the ecological restoration of a tropical shallow lake in a city. Ecol. Enviton. 1, 1-7 (in Chinese). https://doi.org/10.16258/j.cnki.1674-5906.2007.01.001.

Chen, G.R., Liu, X., Ying, W.Y., Guo, X.B., Zhong, P., Liu Z.W., 2007b. The Response of Zooplankton Community to Ecological Restoration in Huizhou West Lake. J. Anhui Agri. Sci. 35(6):1662-1663 (in Chinese). https://doi.org/10.13989/j.cnki.0517-6611.2007.06.049.

Chen, G.R., Zhong, P., Zhang, X.F., Xie, Y.F., LI, C.H., 2008. Zooplankton and its relationship with water quality in Huizhou West Lake. J. Lake Sci. 20(3), 351-356 (in Chinese). https://doi.org/10.18307/2008.0314.

Chen, L.J., Liu, Q., Peng, Z.J., Hu, Z.J., Xue, J.Z., Wang, W., 2011. The variation and analysis of rotifer community structure in and out of crab-net-pen closing in the Yangcheng Lake. J. Fish. China 08, 1247-1257 (in Chinese). https://doi.org/10.3724/SP.J.1231.2011.17369.

Dube, T., Denecker, L., Vuren, J.H.J.V., Wepener, V., Smit, N.J., Brendonck, L., 2017. Spatial and temporal variation of invertebrate community structure in flood-controlled tropical floodplain wetlands. J. Freshwater Ecol. 32, 1-15. https://doi.org/10.1080/02705060.2016.1230562.

Goździejewska, A., Glińska-Lewczuk, K., Obolewski, K., Grzybowski, M., Kujawa, R., Lew, S., Grabowska, M., 2016. Effects of lateral connectivity on zooplankton community structure in floodplain lakes. Hydrobiologia 774, 7-21. https://doi.org/10.1007/s10750-016-2724-8.

Ghadouani, A., Pinel-Alloul, B., Prepas, E.E., 2003. Effects of experimentally induced cyanobacterial blooms on crustacean zooplankton communities. Freshwater Biol. 48, 363-381. https://doi.org/10.1046/j.1365-2427.2003.01010.x.

Huang, X.F., 2000. Investigation, observation and analysis of lake ecology. China Standard Press, Beijing (in Chinese).

Harrison, S., Ross, S.J., Lawton, J.H., 1992. Beta diversity on geographic gradients in Britain. J. Anim. Ecol. 61, 151-158. https://doi.org/10.2307/5518.

Huo, Y.Z., He, W.H., Luo, K., Wang, Y.Y., Zhang Y.J., Tian, Q,T., He, P.M., 2010. Biorem ediation efficiency of applying *Daphnia magna* and submerged plants : A case study in Dishui Lake of Shanghai, Chinese J. Appl. Ecol. 21(2), 495-499 (in Chinese). https://doi.org/10.13287/j.1001-9332.2010.0006.

Hu, C.Y., 1999. The density and diversity of zooplanktons in the succession of five lakes. Acta Hydrobiol. Sin. 3, 3-5 (in Chinese). https://doi.org/10.3321/j.issn:1000-3207.1999.03.004.

Hart, R.C., 2010. Zooplankton distribution in relation to turbidity and related environmental gradients in a large subtropical reservoir: patterns and implications. Freshwater Biol. 24(2), 241-263. https://doi.org/10.1111/j.1365-2427.1990.tb00706.x.

Jiang, X.Z., Du, N.S., 1979. Fauna sinica: crustacean, freshwater cladocera. Science Press, Beijing (in Chinese).

Jernberg, S., Lehtiniemi, M., Uusitalo, L., 2017. Evaluating zooplankton indicators using signal detection theory. Ecol. Indicat. 77, 14-22. https://doi.org/10.1016/j.ecolind.2017.01.038.

Jiang, X.J., Han, B,P., Lin, Q.Q., 2013. Effects of fish stocking density on zooplankton

community structure in reservoirs. J. Hydroecol. 34(01), 30-36 (in Chinese). https://doi.org/10.15928/j.1674-3075.2013.01.002.

Kosten, S., Lacerot, G., Jeppesen, E., Marques, D.M., Nes, E.H., Mazzeo, N., Scheffer, M., 2009. Effects of submerged vegetation on water clarity across climates. Ecosystems 12, 1117-1129. https://doi.org/10.1007/s10021-009-9277-x.

Liu, B.P., Liu, J.H., Wang, S.F., Liang, F., 2018. Relationship between community structure of zooplankton and water quality of a mesotrophic lake wetland. Agr. Biotechnol. 7(4), 236-241+245. https://doi.org/CNKI:SUN:AGBT.0.2018-04-058.

Lin, Q., You, W.H., Xu, F.J., Yu, Q.J., Yu, H.G., 2014. Zooplankton community structure and its relationship with environmental factors in Dishui Lake. Acta Ecol. Sin. 34(23), 6918-6929 (in Chinese). https://doi.org/10.5846/stxb201303060360.

Lin, Z., Wan, Y., Xu, M., Wang, C.C., Wu, Q.L., Zhou, Z.Z., 2018. Metazoan zooplankton community structure and environmental drivers of Digou coal mining subsided lakes in Huainan. J. Lake Sci. 30(01), 171-182 (in Chinese). https://doi.org/10.18307/2018.0117.

Meyer, J., Dahlke, S., Kafka, M., Kerkow, A., Lindner, C., Kube, S., Nawka, B.L., Schubert, H., Schumann, R., Blindow, L., 2018. Submerged vegetation in a shallow brackish lagoon does not enhance water clarity but offers substantial refuge for zooplankton. Aquat. Bot. 154, 1-10. https://doi.org/10.1016/j.aquabot.2018.12.002.

Morse, R.E., Friedland, K.D., Tommasi, D., Stock, C., Nye, J., 2017. Distinct zooplankton regime shift patterns across ecoregions of the u.s. northeast continental shelf large marine ecosystem. J. Marine Syst. 165, 77-91. https://doi.org/10.1016/j.jmarsys.2016.09.011.

Miao, T., Gao, J., Chen, B.H., Liu, Z.W., 2013. The effects of ecological restoration on the community structures of planktonic crustaceans in Huizhou West Lake. Ecol. Sci. 32(3), 324-330 (in Chinese). https://doi.org/CNKI:SUN:STKX.0.2013-03-011.

Nicolle, A., Hansson, L.A., Brodersen, J., Nilsson, P. A., Brönmark, C., 2011. Interactions between Predation and Resources Shape Zooplankton Population Dynamics. PloS One 6(1), e16534. https://doi.org/10.1371/journal.pone.0016534.

Roemmich, D., McGowan, J., 1995. Climatic warming and the Decline of Zooplankton in the California Current. Science 267(5202):1324-1326. https://doi.org/10.1126/science.267.5202.1324.

Špoljar, M., Tomljanović, T., Dražina T., Lajtner J., Štulec, H., Matulić, D., Fressl. J., 2016. Zooplankton structure in two interconnected ponds: similarities and differences. Croat. J. Fish. 74(1), 6-13. https://doi.org/10.1515/cjf-2016-0002.

Shen, J.R., 1979. Fauna sinica: crustacean, freshwater copepoda. Science Press, Beijing (in Chinese).

Simões, N.R., Dias, J.D., Leal, C.M., Braghin, L.S.M., Lansac-Tôha, F.A., Bonecker, C.C., 2013. Floods control the influence of environmental gradients on the diversity of zooplankton communities in a neotropical floodplain. Aquat. Sci. 75, 607-617. https://doi.org/10.1007/s00027-013-0304-9.

Wang, W.C., Sun, S., Zhang, F., Sun, X.X., Zhang, G.T., 2018. Zooplankton community structure, abundance and biovolume in Jiaozhou Bay and the adjacent coastal Yellow Sea during summers of 2005-2012: relationships with increasing water temperature. J. Oceanol.

Limnol. 36(5), 1655-1670. https://doi.org/10.1007/s00343-018-7099-4.

Wang, J.Z., 1961. China's Freshwater Rotifers. Science Press, Beijing (in Chinese).

Wang, H.J., Ding, X.S., Tan, W.J., Zhou Y.L., 2008. Study on the effects of floating plants on eutrophic water bodies. J. Anhui Agri. Sci. 24,10654-10656. https://doi.org/10.13989/j.cnki.0517-6611.2008.24.039.

Wang, C., Wang, L., Deng, D.G., Zhou, Z.Z., 2016. Temporal and spatial variations in rotifer correlations with environmental factors in Shengjin Lake, China. Environ. Sci. Pollut. Res. 23 (8), 8076-8084. https://doi.org/10.1007/s11356-015-6009-y.

Xu, L.L., Xu, W.B., Sun, Q.Y., Zhou, Z.Z., Shen, J., Zhao, X.X., 2008. Flora and vegetation in Shengjin Lake. Plants Sci. J. 3, 264-270 (in Chinese). https://doi.org/10.3969/j.issn.2095-0837.2008.03.010.

Xie, H., Jiang, Z.G., Xia, Z.J., Guo, W.Y., 2018. Functional groups of fish community in the Huayang lake group and their response to enclosure culture. J. Fish. China 42(9), 1399-1407 (in Chinese). https://doi.org/10.11964/jfc.20170610888.

Xu, Z.L., Wang, Y.L., Chen, Y.Q., Shen, H.T., 1995. Ecological study of zooplankton in the largest turbidity zone of the Yangtze River Estuary. J. Fish. Sci. China 2(1), 39-48 (in Chinese). https://doi.org/CNKI:SUN:ZSCK.0.1995-01-003.

Xu, M., 2016. The research on the Community Structure of mezoan zooplaktons in Lake□Jiaoganghu and Lake Huayanghe. Anhui University, Dissertation.

Xue, J.Z., Cai. Z., Fang. W., Wu H.X., Lu. J.H., 2010. Ecological status of plankton in

Kunshan area of Dianshan Lake after aquaculture net dismantling. J. Shanghai Ocean Uni. 19(04), 514-520 (in Chinese). https://doi.org/CNKI:SUN:SSDB.0.2010-04-016.

Xu, D.L., Liu, Z.W., Lei, Z.X., Zeng, Y., Cheng, Y.H., 2005. Advances in the Study of Mechanism of Macrophytes on the Ecological Remediation of Lake. J. Yangtze Uni. 12(02),14-18+104 (in Chinese). https://doi.org/10.3969/j.issn.1673-1409-C.2005.02.004.

Yang, Q.X., Li, W.C., 1996. Effect of intensive pen fish farming on aquatic vegetation and its ecological strategy. Chinese J. Appl. Ecol. 7(1): 83-88 (in Chinese). https://doi.org/ 10.13287 /j.1001-9332.1996.0017.

Yang, L.J., Lu, G.H., Zhu, J.Q., Xu, Z., Jin, C.H., 2014. Characteristics of zooplankton community in Hengshan reservoir and water quality assessment. Acta Hydrobiol. Sin. 38(4), 720-728 (in Chinese). https://doi.org/10.7541/2014.102.

Zhu, W.Z., Zhou, L.Z., 2010. Biodiversity and Conservation in Anqing Floodplain Wetlands. Hefei University of Technology Press, Hefei.

Zhao, C.S., Liu C.M., Zhao, J.H., Xia, J., Yu, Q., Eamus. D., 2013. Zooplankton in highly regulated rivers: Changing with water environment. Ecol. Eng. 58, 323-334. https://doi.org/10.1016/j.ecoleng.2013.07.035.

Zeng. L., He. F., Zhang Y., Liu B.Y., Dai Z.G., Zhou, Q.H., Wu Z.B., 2018. Size-dependent responses of zooplankton to submerged macrophyte restoration in a subtropical shallow lake. J. Oceanol. Limnol. 36(2), 376-384. https://doi.org/CNKI:SUN:HYFW.0.2018-02-018.

Zhang, K., Jiang, F.Y., Chen, H., Dibar, D.T., Wu, Q.L., Zhou, Z.Z., 2019. Temporal and spatial variations in zooplankton communities in relation to environmental factors in four floodplain lakes located in the middle reach of the Yangtze River, China. Environ. Pollut. 251, 277-284. https://doi.org/10.1016/j.envpol.2019.04.139.

Zhang, Z.S., Huang, X.F., 1991. Study methods of freshwater plankton. Science Press, Beijing (in Chinese).

Zhong, J.X., Lin, X.T., Xu, Z.N., J, X.Y., 2001. The top-down effect of fish stocking on the freshwater environment (A review). J. Jinan Uni. 05, 131-136 (in Chinese). https://doi.org/10.3969/j.issn.1000-9965.2001.05.026.

Zheng, W., Sugie, J., 2019. Global asymptotic stability and equiasymptotic stability for a time-varying phytoplankton-zooplankton-fish system. Nonlinear Anal-Real 46, 126-136. https://doi.org/10.1016/j.nonrwa.2018.09.015.

Zhang, M., Liu, R.J., Zhang, Z.H., Cao, Z.H., Deng, J.Z., Wang, H.W., 2018. Effects of different environmental factors on cladocera communities in lakes. Hebei Fish. 08, 43-45 (in Chinese). https://doi.org/10.3969/j.issn.1004-6755.2018.08.013.

Zhang Yao, 2019. The important role of plankton in fish culture. Agr. Dev. Equipments 1, 147 (in Chinese). https://doi.org/CNKI:SUN:NJJY.0.2019-01-099.

Zeng, H.Y., Zhong, P., Zhao, X.F., Li, C., He, X.L., Liu, Z.W., 2016. Response of metazoan zooplankton communities to ecological restoration in a tropical shallow lake. J. Lake Sci. 28(01), 170-177 (in Chinese). https://doi.org/10.18307/2016.0120.

Captions

Fig 1. Distribution map of sampling sites in the Huayanghe Lakes.

Fig 2. Distribution map of main aquatic vegetation in the Huayanghe Lakes was in the summer of 2015(a) and 2018(b). Abbreviations was used in the diagram: Mv: *Myriophyllum verticillatum*; Cd: *Ceratophyllum demersum*; Vn: *Vallisneria natans*; Pl: *Polygonum lapathifolium*; Po: *Polygonum orientale*; Sn: *Salvinia natans*; Pa: *Phragmites australis*; Zc: *Zizania caduciflora*; Ec: *Eichhornia crassipes*; Ef: *Euryale ferox*; Nn: *Nelumbo nucifera*; Ti: *Trapa incisa*; Pw: *Potamogeton wrightii*; Np: *Nymphoides peltatum*.

Fig 3. The diversity index of metazooplankton taxa in the Huayanghe Lakes collected in different seasons.

Fig 4. Temporal variation of metazooplankton density in the Huayanghe Lakes.

Fig 5. Distribution of metazooplankton density at three studied lakes. (The plot is expressed in terms of the annual average metazooplankton density, the unit is ind./L).

Fig 6. Biplot diagram for Redundancy analysis between major metazooplankton species (solid arrowhead) and environment factors (hollow arrow with line) in the Huayanghe Lakes. Abbreviations was used in the diagram: *Kc*: *Keratella cochlearis*, *Pt*: *Polyarthra trigla*, *Kq*: *Keratella quadrata*, *Bc*: *Branchionus clycifolrus*, *Tp*: *Trichocerca pusilla*, *Ml*: *Monostyla lunaris*, *Kv*: *Keratella valga*, *Ba*: *Brachionus angularis*, *Bf*: *Brachionus forficulav*, *Bb*: *Branchionus budapestiensis*, *Ch*: *Conochilus hippocrepi*, *Ae*: *Ascomorpha ecaudis*, *As*: *Ascomorpha saltans*, *Ap*: *Asplachna priodonta* Gosse, *Th*: *Thermocyclops hyalinus*, *Mv*: *Microcyclops vaticans*, *Cv*: *Cyclops vicinus*, TZD: Annual mean density.

Fig 1

Click here to access/download;Figure;Fig 1.tif

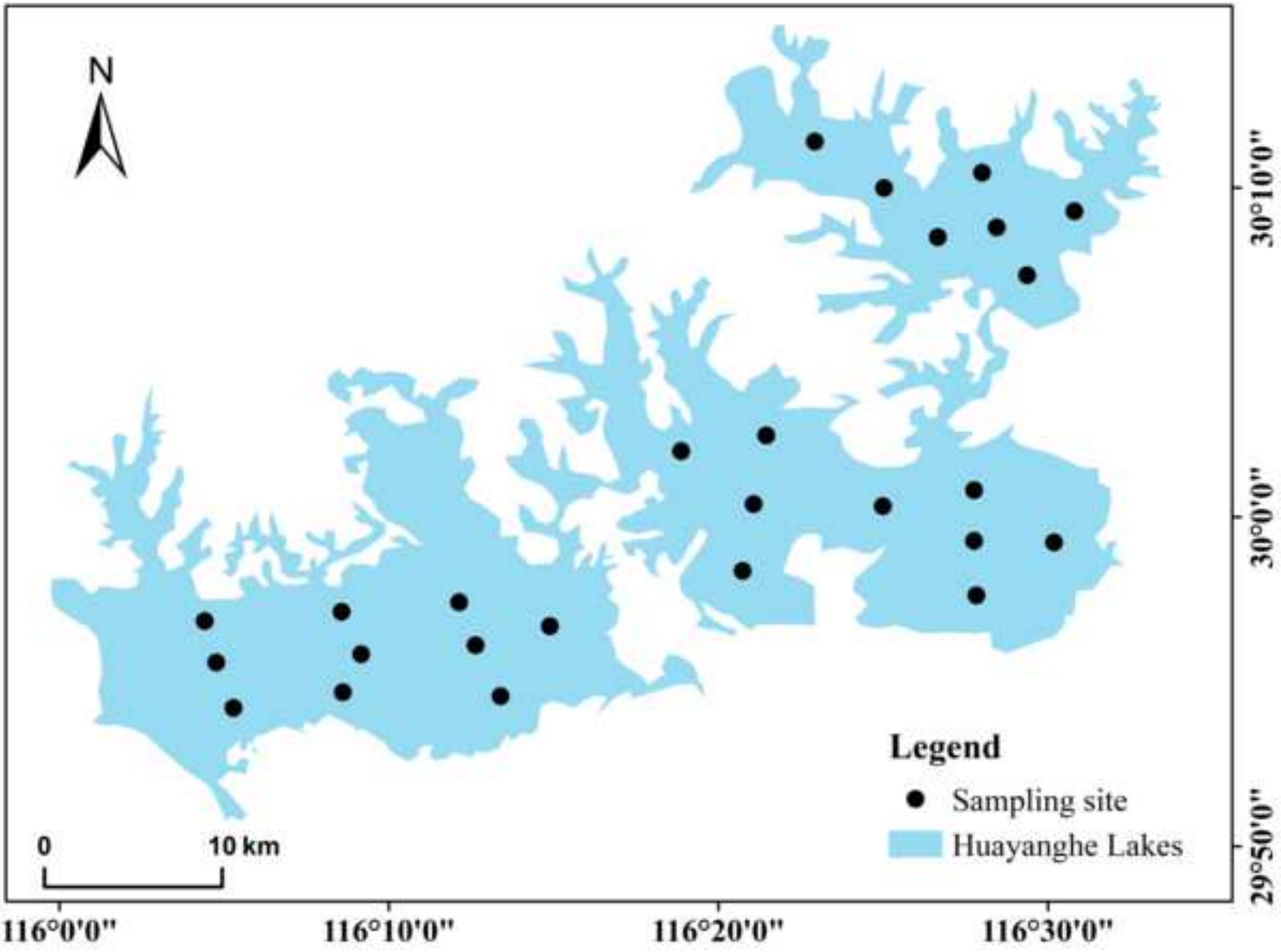

Fig 2

Click here to access/download;Figure;Fig 2.tif

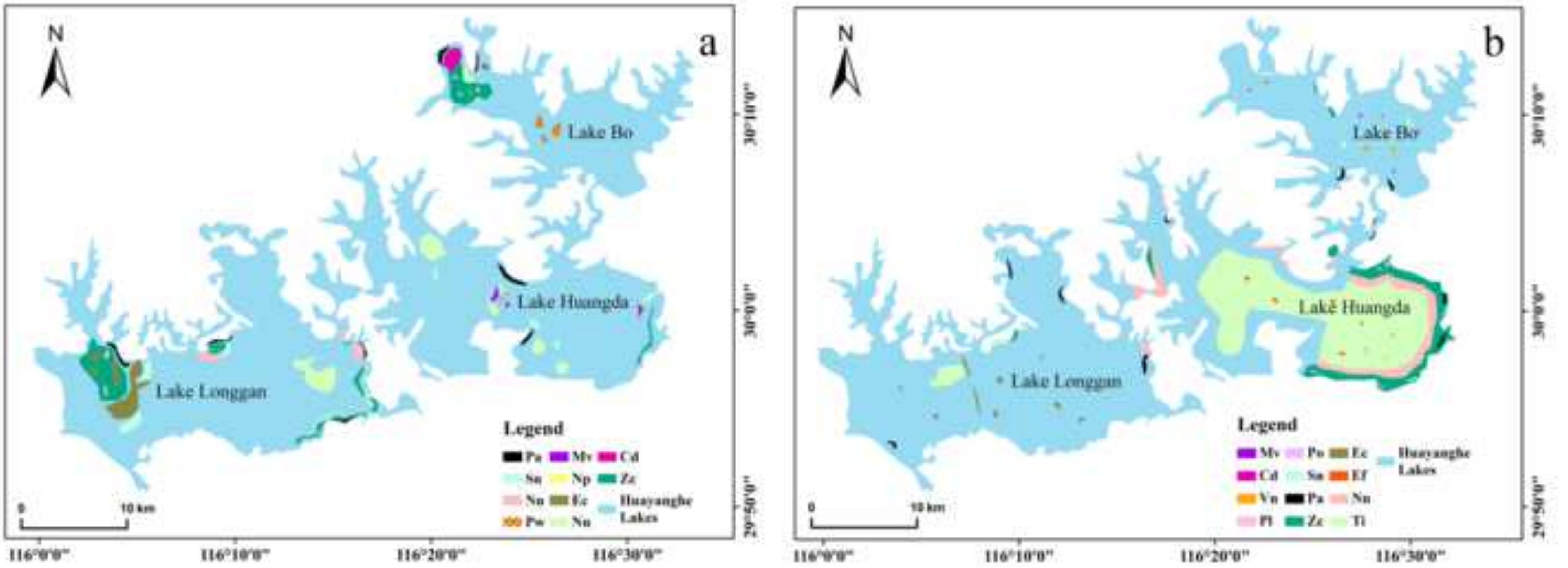

Click here to access/download;Figure;Fig 3.tif

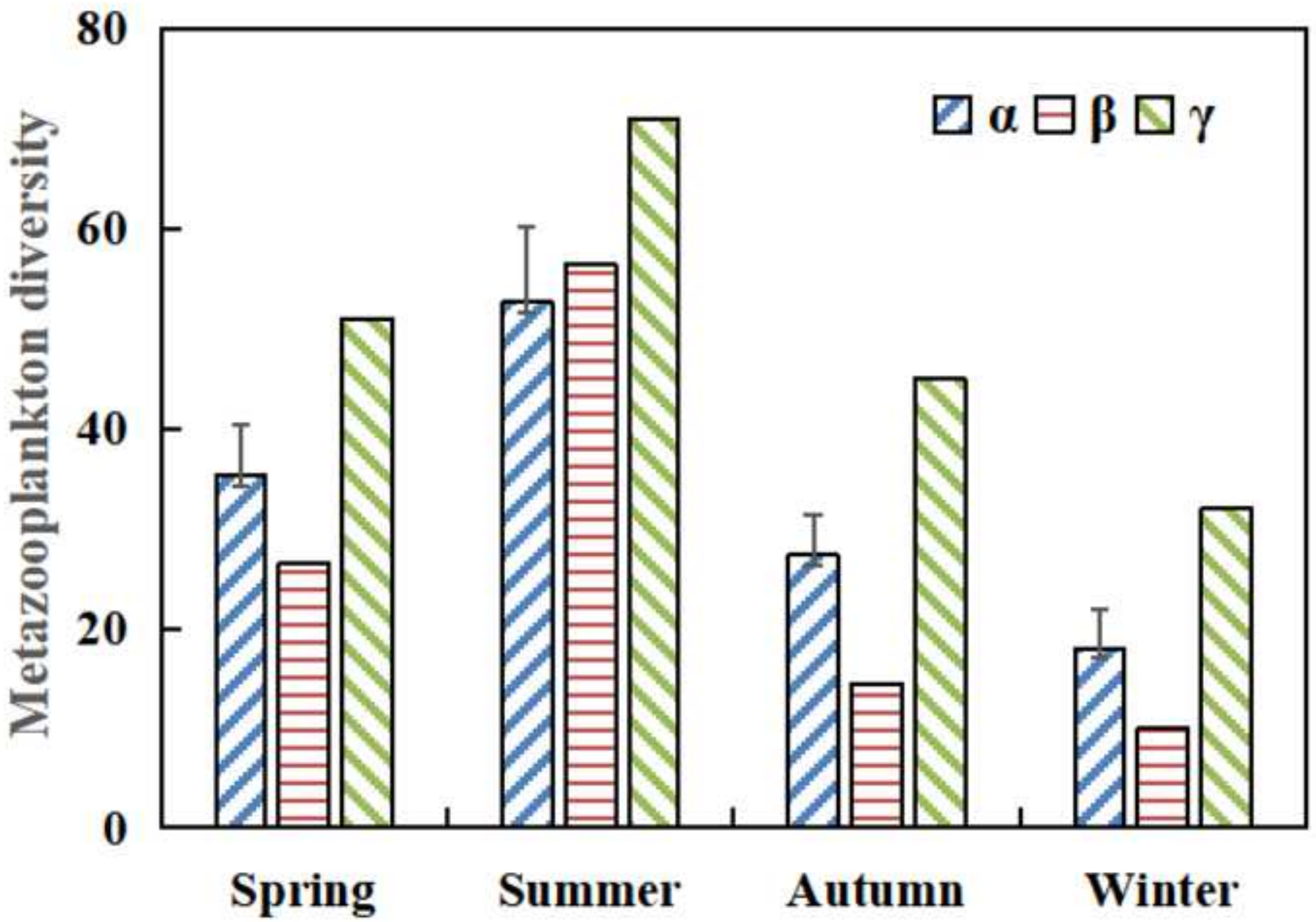

Fig 4

Click here to access/download;Figure;Fig 4.tif

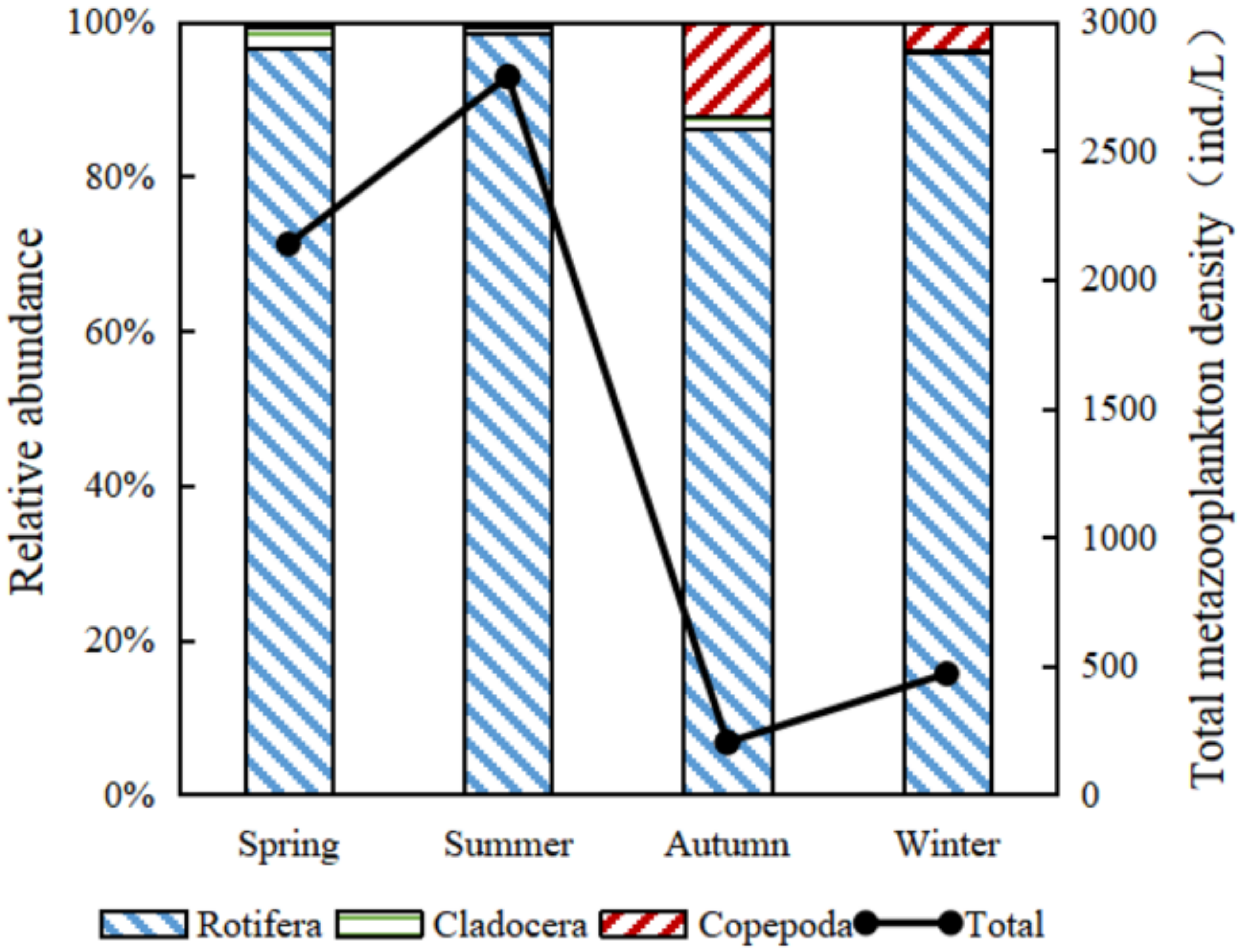

Fig 5

Click here to access/download;Figure;Fig 5.tif

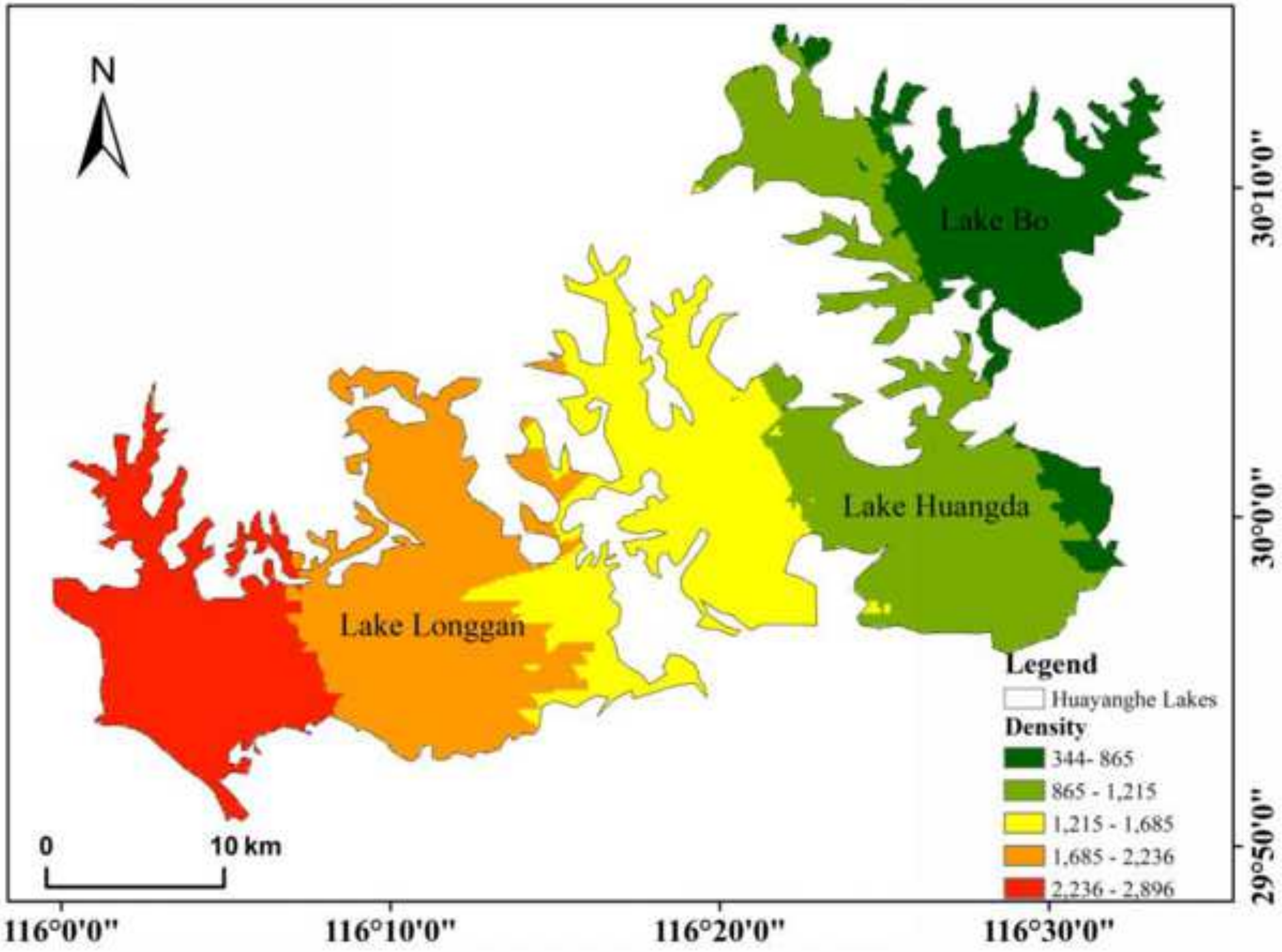

Fig 6

Click here to access/download;Figure;Fig 6.tif

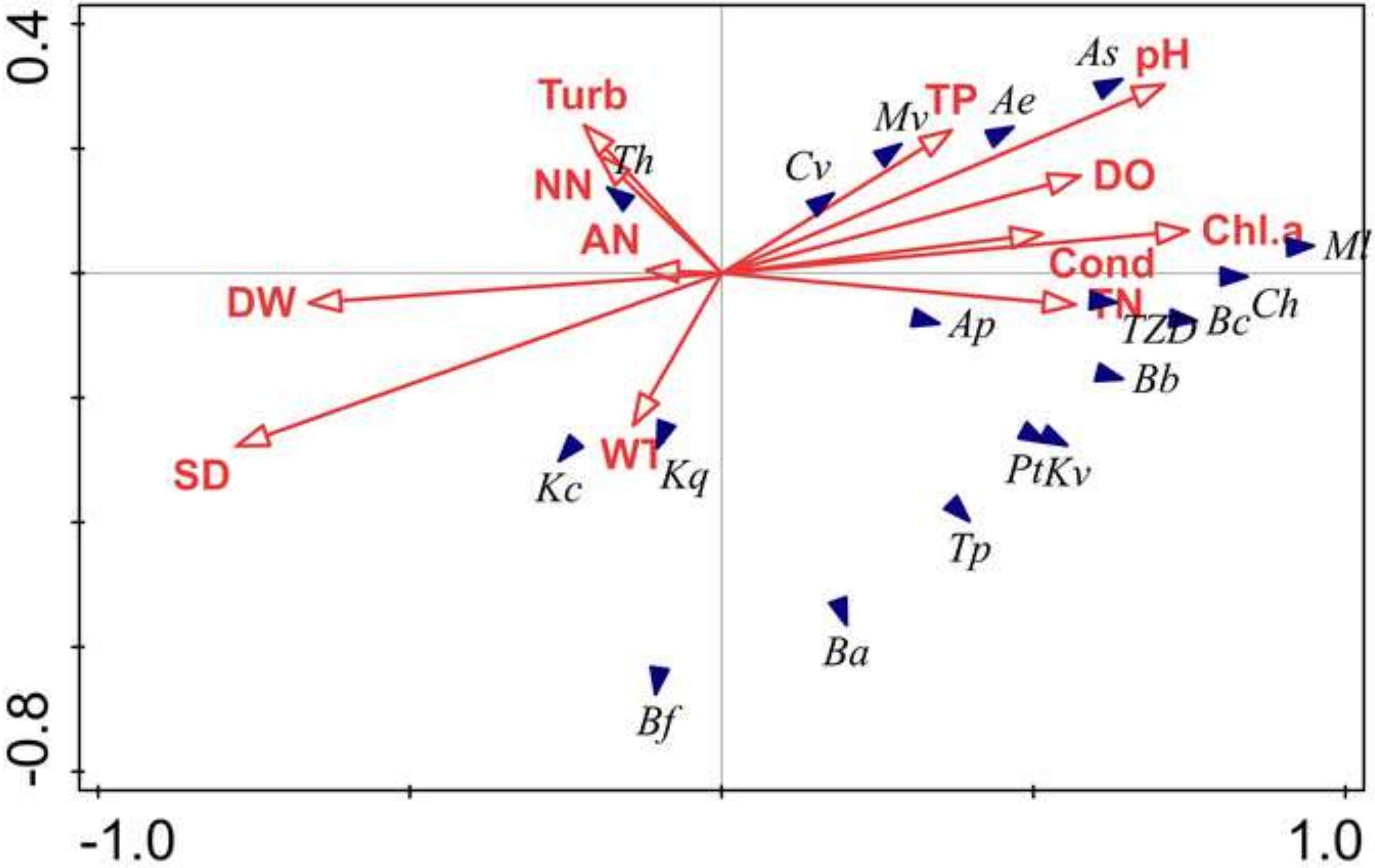

Supplement: Attachment — Submitted filename: PONE-D-21-05633_reviewer.pdf [file pone.0252100.s002.pdf]
