# Supplementary material for: The impact of withdrawing aquaculture facilities on metazooplankton communities in the lakes are connected to the Yangtze River, China
Source: PLoS One. 2021 May 26;16(5):e0252100. doi: 10.1371/journal.pone.0252100 (PMC8153418; doi:10.1371/journal.pone.0252100)
Supplement: Attachment — Submitted filename: Response to Reviewers.doc [file pone.0252100.s003.doc]

Dear Editor and reviewers,

On behalf of my co-authors, we thank you very much for giving us an opportunity to revise our manuscript, we appreciate editor and reviewers very much for their positive and constructive comments and suggestions on our manuscript. We have studied editor and reviewers' comments carefully and have made revision which marked in blue in the paper.

I modified the way of inserting references and sorted the documents according to the requirements of this journal, so the use of some documents in the main text will change. If this has caused some trouble when you re-review it, I am deeply sorry.

We would like to express our great appreciation to you and reviewers for comments on our paper. Looking forward to hearing from you.

Thank you for your time.

Sincerely yours,

Yixing Zhang, Yutao Wang, Zhongze Zhou

**Journal Requirements/Response to Editor**

1. Please ensure that your manuscript meets PLOS ONE's style requirements, including those for file naming. The PLOS ONE style templates can be found at

https://journals.plos.org/plosone/s/file?id=wjVg/PLOSOne_formatting_sample_main_body.pdf and

[https://journals.plos.org/plosone/s/file?id=ba62/PLOSOne_formatting_sample_title_authors_affiliations.pdf](https://mail.ahu.edu.cn/js6/read/about:blank)

**Answer:**

Thank you for your kind suggestions. I have modified the format of my article according to the format of the paper recently published in this journal.

2. In your Methods section, please provide additional location information of the study sites, including geographic coordinates for the data set if available.

**Answer:**

Thank you for your kind suggestions. I added additional location information of the study sites. And the following table is the data set of the geographic coordinates of my study sites, and I put it in the supporting file.

**Lines 79-81** in the revised MS: “Huayanghe Lakes (116°00"E-116°33"E, 29°52"N-30°58"N) lie on the north bank of the Yangtze River in Anhui Province, China. The lakes have a surface area of 580 ± 115.8 km^2^. The average water level is 14.2 m with the average water depth of 4.0 m. ”

Tab geographic coordinates of the study sites

|  | longitude | latitude |
| --- | --- | --- |
| L2 | 116.07347 | 29.9472 |
| L3 | 116.07931 | 29.92637 |
| L4 | 116.08807 | 29.90331 |
| L5 | 116.14265 | 29.95196 |
| L6 | 116.15261 | 29.93054 |
| L7 | 116.14334 | 29.9112 |
| L8 | 116.21046 | 29.935 |
| L9 | 116.22316 | 29.90926 |
| L10 | 116.20222 | 29.95672 |
| L11 | 116.24805 | 29.94467 |
| H12 | 116.314487 | 30.033285 |
| H13 | 116.35757 | 30.04116 |
| H14 | 116.35122 | 30.00653 |
| H15 | 116.34556 | 29.97263 |
| H16 | 116.41645 | 30.00549 |
| H17 | 116.46263 | 30.01352 |
| H18 | 116.462631 | 29.987799 |
| H19 | 116.46383 | 29.96029 |
| H20 | 116.50314 | 29.98706 |
| S0 | 116.51344 | 30.15463 |
| S1 | 116.46675 | 30.17422 |
| S2 | 116.47413 | 30.14646 |
| S3 | 116.48941 | 30.12226 |
| S4 | 116.44426 | 30.14156 |
| S5 | 116.41714 | 30.16635 |
| S6 | 116.38212 | 30.18995 |

3. In your Methods section, please provide additional information regarding the permits you obtained for the work. Please ensure you have included the full name of the authority that approved the field site access and, if no permits were required, a brief statement explaining why.

**Answer:**

Thank you for your comment on my paper.

No specific permissions were required for the described field studies. The lakes studied are not privately owned or protected in any way and the field studies did not involve endangered or protected species.

4. We suggest you thoroughly copyedit your manuscript for language usage, spelling, and grammar. If you do not know anyone who can help you do this, you may wish to consider employing a professional scientific editing service.

**Answer:**

Thank you for your kind suggestions. I have corrected my paper through others' advice. And it's shown in the Acknowledgements section of my manuscript.

**Lines 411-414** in the revised MS: “...We would also like to thank Dr. Chunlin Li and Xia Wan for their advice on the paper. We would like to thank Marci Baun from University of California, Los Angeles for editing the paper....”

5. Our internal editors have looked over your manuscript and determined that it is within the scope of our Freshwater Ecosystems Call for Papers. This collection of papers is headed by a team of Guest Editors for PLOS ONE (https://collections.plos.org/s/freshwater-ecosystems). The Collection will encompass a diverse range of research articles on freshwater ecology, including lake ecology. Additional information can be found on our announcement page: https://collections.plos.org/s/freshwater-ecosystems.

If you would like your manuscript to be considered for this collection, please let us know in your cover letter and we will ensure that your paper is treated as if you were responding to this call. If you would prefer to remove your manuscript from collection consideration, please specify this in the cover letter.

**Answer:**

Thanks for the magazine's recognition of my research and manuscript. In the cover letter, I expressed my willingness to have my manuscript considered by your magazine.

6. We note that the grant information you provided in the ‘Funding Information’ and ‘Financial Disclosure’ sections do not match.

When you resubmit, please ensure that you provide the correct grant numbers for the awards you received for your study in the ‘Funding Information’ section.

**Answer:**

Thank you for your kind suggestions. I will fix it when I resubmit it.

7. In your Data Availability statement, you have not specified where the minimal data set underlying the results described in your manuscript can be found. PLOS defines a study's minimal data set as the underlying data used to reach the conclusions drawn in the manuscript and any additional data required to replicate the reported study findings in their entirety. All PLOS journals require that the minimal data set be made fully available. For more information about our data policy, please see <http://journals.plos.org/plosone/s/data-availability.>

**Answer:**

Thank you for your kind suggestions. I have added the drawing data to the attached file and uploaded it.

8.You may seek permission from the original copyright holder of Figures 1, 2, 5 to publish the content specifically under the CC BY 4.0 license.

We recommend that you contact the original copyright holder with the Content Permission Form (http://journals.plos.org/plosone/s/file?id=7c09/content-permission-form.pdf) and the following text: ......

**Answer:**

Thank you for your kind suggestions. Fig 1, 2, and 5 were drawn by myself using software, so we didn't need to ask for a copyright license from them. The original picture file has been saved as .Ai format, and will be uploaded to the supporting information as an attachment, this editable vector file can prove the original nature of our images. If you have any other requirements/questions, please let us know in time and we will provide further documents required.

**Response to Reviewer #1**

Introduction

Original: in aquatic ecosystems (Zhang, 2019).

Suggestion: in aquatic ecosystems (Zhang, 2019). And, the zooplankton have long been noted as a secondary producer by occupying almost middle positions of the food chain (Tugyan and Bozkurt, 2019). Compared with other aquatic….

You can add the section as literature in yellow. International literature contribution

Tugyan, C., Bozkurt, A., 2019. A Study on Zooplankton Fauna and Some Water Quality Parameters of Kozan Dam Lake (Adana, Turkey). Journal of Limnology and Freshwater Fisheries Research 5(3): 147-158.

**Answer:**

Thank you for your detailed suggestions. I have added it to my manuscript.

**Lines 41-43** in the revised MS: “...in aquatic ecosystems [6]. And, the zooplankton have long been noted as a secondary producer by occupying almost middle positions of the food chain [7]. Compared with other aquatic animals...”

**Line 434-436** was the matching literature in the revised MS: "7. Tugyan C, Bozkurt A. A Study on Zooplankton Fauna and Some Water Quality Parameters of Kozan Dam Lake (Adana, Turkey). J Limnol Freshw Fish Res. 2019; 5(3): 147-158. https://doi.org/10.17216/limnofish.538344."

Original: 68 line (http://www.susongbbs.com/1408365-1-1.html)

69 line (http://www.ahmhxc.com/tongjigongbao/14909_2.html).

Suggestion: these links show in Chinese. It doesn't make sense for the reader. Because not everyone knows Chinese. Please remove the link.

**Answer:**

Thank you for your constructive suggestions. I removed the links.

**Line 65-66** in the revised MS: " Susong County’s water production dropped sharply from 90400 t in 2017 to 83900 t in 2018."

Sampling collection and treatment

Original 106 line and Du (1979), and Shen (1979).
Suggestion: Why did you only use Chinese researchers in the reference books you use for species diagnosis?
Please include here other authors from other countries with international books. For example; Koste 1978……..
(Koste, W., 1978. Rotatoria, Die Rädertiere Mitteleuropas Ein Bestimmungswerk, Begründet von Max Voigt Überordnung Monogononta. I Textband. Gebrüder Borntraeger, Berlin, Stuttgart. 672P and II Textband. 234P.)

**Answer:**

Thank you for your detailed suggestions. I have added it to my manuscript. But I'm so sorry, I can't find any other reference books. I will continue to work hard in the future study.

**Line 106-107** in the revised MS: "... by Wang [18], Koste [19], Jiang and Du [20], and Shen [21]."

**Line 469-471** was the matching literature in the revised MS: "19. Koste W. Rotatoria, Die Rädertiere Mitteleuropas Ein Bestimmungswerk, Begründet von Max Voigt Überordnung Monogononta. 1978; I Textband. Gebrüder Borntraeger, Berlin, Stuttgart. 672P and II Textband. 234P."

Results

Original 161 line summer, Ass. N. nucifera and Ass. Z. caduciflora

Suggestion: Ass. Nelumbo nucifera and Ass. Z. caduciflora (write the expansion of the species)

**Answer:**

Thank you for your detailed suggestions. As suggested, we replaced "Ass. N. nucifera and Ass. Z. caduciflora" with "Ass. Nelumbo nucifera and Ass. Zizania caduciflora".

**Line 162** in the revised MS: "Ass. *Nelumbo nucifera* and Ass. *Zizania caduciflora*..."

Original 162 line waters of Lake Bo; Ass. T. incisa

Suggestion: waters of Lake Bo; Ass. T. incisa (write the expansion of the species)

**Answer:**

Thank you for your detailed suggestions. As suggested, we replaced "Ass. T. incisa" with "Ass. Trapa incisa".

**Line 163** in the revised MS: "...waters of Lake Bo; Ass. *Trapa incisa*..."

Original 163 line Ass. Potamogeton Wrightii

Suggestion: Ass. Potamogeton wrightii

**Answer:**

Thank you for your detailed suggestions. As suggested, we replaced "Ass. *Potamogeton Wrightii*" with "Ass. *Potamogeton wrightii*".

**Line 165** in the revised MS: "Ass. *Potamogeton wrightii* ..."

Original 174 line There were small areas of T. incisa
Suggestion: There were small areas of T. incisa
Original 194 line Trichocerca pusilla, K. cochlearis, P. trigla and
Suggestion: Trichocerca pusilla, K. cochlearis, P. trigla and (write the expansion of the species)
**Answer:**

Thank you for your detailed suggestions. As suggested, we wrote the expansion of the species.

**Line 174-175** in the revised MS: "There were small areas of *Trapa incisa*..."

**Line 195** in the revised MS: "...*Trichocerca pusilla*, *Keratella cochlearis*, *Polyarthra trigla* and..."

Original 200 line In addition, K. quadrata, P. trigla, B. angularis,
Suggestion: In addition, K. quadrata, P. trigla, B. angularis, (write the expansion of the species)
**Answer:**

Thank you for your detailed suggestions. As suggested, we wrote the expansion of the species.

**Line 201-202** in the revised MS: "In addition, *Keratella quadrata*, *Polyarthra trigla*, *Brachionus angularis*,..."

Original 230 line M. lunaris, B. forficula, and
Suggestion: M. lunaris, B. forficula, and (write the expansion of the species)
**Answer:**

Thank you for your detailed suggestions. As suggested, we wrote the expansion of the species. At the same time, I found the errors of the species and corrected them. Apologies for this oversight.

**Line 230** in the revised MS: "*Monostyla lunaris*, *Conochilus hippocrepis* and,..."

Original 231 line correlated with Chl α, TN, and Cond, while T. hyalinus
Suggestion: correlated with Chl α, TN, and Cond, while T. hyalinus (write the expansion of the species)
**Answer:**

Thank you for your detailed suggestions. As suggested, we wrote the expansion of the species.

**Line 230-232** in the revised MS: "Chl α, TN, and Cond..., but negatively impacted *Thermocyclops hyalinus*..."

Original 233 line the main influencing factor for K. cochlearis and K. quadrata. A. saltans, A. ecaudis
Suggestion: the main influencing factor for K. cochlearis and K. quadrata. A. saltans, A. ecaudis (write the expansion of the species)
Original 234 line T. hyalinus were positively
Suggestion: T. hyalinus were positively
**Answer:**

Thank you for your detailed suggestions. As suggested, we wrote the expansion of the species. At the same time, I found the errors of the species and corrected them. Apologies for this oversight.

**Line 233** in the revised MS: "WT was the main influencing factor for *Keratella cochlearis* and *Keratella quadrata.*"

**Line 233-235** in the revised MS: "TP and pH had a positive influence on *Ascomorpha saltans*, *Ascomorpha ecaudis*, *Microcyclops vaticans* and *Cyclops vicinus*..."

Original 283 line We observed small areas of T. incisa and 283 scattered floating E. crassipes
Suggestion: We observed small areas of T. incisa and 283 scattered floating E. Crassipes (write the expansion of the species)
**Answer:**

Thank you for your detailed suggestions. As suggested, we wrote the expansion of the species.

**Line 284-285** in the revised MS: "We observed small areas of *Trapa incisa* and scattered floating *Eichhornia crassipes*..."

Original 331 line Sida erystallina and
Suggestion: Sida crystallina and

**Answer:**

Thank you for your detailed suggestions. As suggested, we replaced "*Sida erystallina*" with "*Sida crystallina*".

**Line 330** in the revised MS: "*Sida crystallina* and ..."

Original 332 line (Daphnia from the original only
Suggestion: (Daphnia from the original only
**Answer:**

Thank you for your detailed suggestions. As suggested, we replaced "*D. cucullata*" with "*Daphnia cucullata*".

**Line 331-332** in the revised MS: "Daphnia from the original only *Daphnia cucullata*..."

Original 337 line as S. erystallina and
Suggestion: as S. crystallina and

**Answer:**

Thank you for your detailed suggestions. As suggested, we replaced "*S. erystallina*" with "*Sida crystallina*".

**Line 336** in the revised MS: "..., such as *Sida crystallina* and..."

Original 337 line dominated by the indicator species of the middle fouling zone, such as B. budapestiensis, ,
Suggestion: dominated by the indicator species of the middle fouling zone, such as B. budapestiensis, B. forficula (write the expansion of the species)
**Answer:**

Thank you for your detailed suggestions. As suggested, we wrote the expansion of the species.

**Line 340-342** in the revised MS: "... the indicator species of the middle fouling zone, such as *Branchionus budapestiensis*, *Brachionus forficula* and *Keratella cochlearis...*"

Original 354 line New species, such as D. magna, D. hyalina, S. erystallina,
Suggestion: New species, such as D. magna, D. hyalina, S. crystallina,

**Answer:**

Thank you for your detailed suggestions. As suggested, we replaced "*S. erystallina*" with "*S. crystallina*". And we wrote the expansion of the species.

Line **353-354** in the revised MS: "We observed some species, such as *Daphnia magna*, *Daphnia hyalina*, *Sida crystallina*, ..."

Original 269 line (Meyer et al., 2019).
Suggestion: (Meyer et al., 2018). 2018 written in references. Correct in the text as above.

Citations in the text should be given in order according to the years. Please apply.

**Answer:**

Thank you for your detailed suggestions. I have corrected them according to the requirements of this magazine for references insertion and references ordering.

**Line 269** in the revised MS: "... fixed sediments and mud, and reduces wind and wave action [36]."

Original: 35 line affecting the safety of the entire lake wetland ecosystem (Zhu et al., 2010).
Suggestion: Include this resource in the References section. Not seen in references.
**Answer:**

Thank you for your detailed suggestions. As suggested, The matching literature was added in the appropriate place.

**Line 35-36** in the revised MS: "...affecting the safety of the entire lake wetland ecosystem [2]."

**Line 421-422** was the matching literature in the revised MS: "2. Zhu WZ, Zhou LZ. Biodiversity and Conservation in Anqing Floodplain Wetlands. Hefei University of Technology Press, Hefei; 2010."

Original: 284 line The study by Jiang et al. (2008) mentioned that
Suggestion: Include this resource in the References section. Not seen in references.
**Answer:**

Thank you for your detailed suggestions. I used the wrong literature. Forgive me for my oversight. As suggested, The matching literature was added in the appropriate place.

**Line 285-286** in the revised MS: "The study by Wang et al. [41] mentioned..."

**Line 531-533** was the matching literature in the revised MS: "41. Wang HJ, Ding XS, Tan WJ, Zhou YL. Study on the effects of floating plants on eutrophic water bodies. J Anhui Agri Sci. 2008; 24: 10654-10656.

https://doi.org/10.13989/j.cnki.0517-6611.2008.24.039. "

Original: 328 line Zeng Lei et al. (2018) reported in their study
Suggestion: Include this resource in the References section. Not seen in references.
**Answer:**

Thank you for your detailed suggestions. As suggested, The matching literature was added in the appropriate place.

**Line 326-328** in the revised MS: "This is consistent with the results of other scholars[8, 12], ..."

**Line 450-452** was the matching literature in the revised MS: "12. Zeng L, He F, Zhang Y, Liu BY, Dai ZG, Zhou QH, Wu ZB. Size-dependent responses of zooplankton to submerged macrophyte restoration in a subtropical shallow lake. J Oceanol Limnol. 2018; 36(2): 376-384. https://doi.org/CNKI:SUN:HYFW.0.2018-02-018."

Notes: reference section shortcomings are noted below.
442 line Huang, X.F., 2000. Investigation, observation and analysis
(This article is not cited in the text. Check it in the text)
**Answer:**

Thank you for your detailed suggestions. As suggested, The matching literature was added in the appropriate place.

**Line 86-87** in the revised MS: "...water environment monitoring part of "The Environmental Monitoring Technical Specifications" [16] ..."

**Line 464-465** was the matching literature in the revised MS: "16. Huang XF. Investigation, observation and analysis of lake ecology. China Standard Press, Beijing (in Chinese); 2000."

506 line Wang, H.J., Ding, X.S., Tan, W.J., Zhou Y.L., 2008. Study on the effects of floating plants (This article is not cited in the text. Check it in the text)
**Answer:**

Thank you for your detailed suggestions. I used the wrong literature. Forgive me for my oversight. As suggested, The matching literature was added in the appropriate place.

**Line 285-286** in the revised MS: "The study by Wang et al. [41] mentioned..."

**Line 531-533** was the matching literature in the revised MS: "41. Wang HJ, Ding XS, Tan WJ, Zhou YL. Study on the effects of floating plants on eutrophic water bodies. J Anhui Agri Sci. 2008; 24: 10654-10656.

https://doi.org/10.13989/j.cnki.0517-6611.2008.24.039. "

515 line Xie, H., Jiang, Z.G., Xia, Z.J., Guo, W.Y., 2018. Functional groups of fish community in (This article is not cited in the text. Check it in the text)
**Answer:**

Thank you for your detailed suggestions. I have deleted this document. Forgive me for my oversight.

535 Zhu, W.Z., Zhou, L.Z., 2010. Biodiversity and Conservation in Anqing Floodplain (This article is not cited in the text. Check it in the text)

535 Zhu, W.Z., Zhou, L.Z., 2010. Biodiversity and Conservation in Anqing Floodplain (This article is not cited in the text. Check it in the text) Include this resource in the References section. Not seen in references.
**Answer:**

Thank you for your detailed suggestions.

**Line 35-36** in the revised MS: "...affecting the safety of the entire lake wetland ecosystem [2]."

**Line 411-412** was the matching literature in the revised MS: "2. Zhu WZ, Zhou LZ. Biodiversity and Conservation in Anqing Floodplain Wetlands. Hefei University of Technology Press, Hefei; 2010."

**Response to Reviewer #2**

I found this research manuscript very interesting and I believe it has scientific impacts. The objectives are very clearly defined. Enough dedications and efforts are observed in results and findings. Authors tried a lot to make this research statistically sound and presentable. Therefore, I have some critical comments on it. Firstly, The discussion need to improve a lot. Please consider my comments very positively. If you reform and rewrite the discussion...then this manuscript could be a master piece. Please avoid gerund sentence (starting with verb+ing). Please read more related articles and rewrite your discussion. Please read and cite more related references in discussion. I expected a conclusion but I didn't find it. Please write a nice conclusion. After all of your revision activities, at the last please write the abstract again. Please follow professional English to make your article more convenience to readers. Trust me you will get more positive feedback on your article if you consider these comments.

If possible try to contact a person who help you in professional English. Please don't feel hesitate on it. I found your research article is very helpful to us but need to be more readable and clear. Hopefully, you got my messages.

**Answer:**

Thank you so much for your positive comments and constructive suggestions. We sincerely appreciate your spending time to review the manuscript. Next, I'll respond to your questions one by one.

First of all, I added the conclusion to the article.

**Line 393-407** in the revised MS:

"5. Conclusion

This study showed that the withdrawing of aquaculture facilities in the Huayanghe Lakes accelerated the restoration and reconstruction of aquatic plants, promoted the improvement of water quality and increased the γ diversity of metazooplankton, especially in Lake Bo with better restoration of aquatic plants. In order to effectively mitigate water eutrophication and improve biodiversity, we recommend that aquaculture facilities be completely dismantled and aquatic plants restored. Studies have shown that the restoration of aquatic plants improves the transparency of water bodies, increases the diversity of metazooplankton species, and increases the density of metazooplankton. However, in the early stage of aquatic plants recovery, the main plants were floating-leaved plants, floating plants and emergent plants, while submerged plants had relatively poor recovery. In order to restore a more stable ecosystem, artificial planting of submerged plants can be combined with management and maintenance to optimize the structure of aquatic vegetation, protect the diversity of aquatic plants and ultimately reduce the adverse effects of lake eutrophication."

1. **Line 1-2** “Title might be rearranged like "The impact of withdrawing aquaculture facilities on metazooplankton communities in the lakes are connected to the Yangtze River, China".”

**Answer:**

Thank you for your detailed suggestions. As suggested, the title has been revised.

**Line 1-2** in the revised MS: "The impact of withdrawing aquaculture facilities on metazooplankton communities in the lakes are connected to the Yangtze River, China"

2. Please rewrite the abstract after reforming the discussion part mainly. In abstract only results are focused. I expect some of your recommendations and opportunities for further researches also here.

**Answer:**

Thank you for your detailed suggestions. I have rewritten my abstract section in my manuscript.

**Line 10-23** in the revised MS: "The withdrawal of aquaculture facilities has an important impact on the aquatic ecosystem of the lakes connected to the Yangtze River. In order to elucidate the response mechanism of metazooplankton to the changes in water environment after the removal of aquaculture facilities, we collected metazooplankton samples and investigated the water environment in the Huayanghe Lakes from the summer of 2018 to the spring of 2019. Aquatic plants recovered quickly, and water eutrophication was relieved, especially in Huangda Lake, followed by Bo Lake. During our study, the highest regional (γ) diversity was 71 in summer, while the lowest was 32 in winter. Species turnover in space (β diversity) varied between 10.01 and 56.52, which was highest in summer. Based on redundancy analysis, environmental factors such as transparency, Chl α, water temperature and water depth, had greatly effects on the metazooplankton community structure. The results showed that the restoration of aquatic plants increased species diversity and metazooplankton density. Our study provides a data basis for lakes restoration and a scientific basis for the management and protection of lakes water ecosystem."

**Introduction**

3. **Line 36-39** “Metazooplankton, mainly composed of rotifera, cladocera and copepoda, feed mainly on smaller phytoplankton, protozoa, bacteria and some organic detritus, and are important primary consumers in aquatic ecosystems. Metazooplankton is also an economic aquatic animal, which can be used as important food for fish and other large aquatic organisms.”

Lack of references and citations. Please read carefully and add related references.

**Answer:**

Thank you for your detailed suggestions. I added the appropriate references.

**Line 36-40** in the revised MS: "Metazooplankton, mainly composed of rotifera, cladocera and copepoda, feed mainly on smaller phytoplankton, protozoa, bacteria and some organic detritus, and are important primary consumers in aquatic ecosystems [3-4]. Metazooplankton is also an economic aquatic animal, which can be used as important food for fish and other large aquatic organisms [5]."

**Line 423-425** in the revised MS: "3. Mcclain CR, Barry JP. Habitat heterogeneity, disturbance, and productivity work in concert to regulate biodiversity in deep submarine canyons. Ecology. 2010; 91(4): 964-976."

**Line 426-427** in the revised MS: "4. Cavan EL, Henson SA, Anna B, et al. Role of zooplankton in determining the efficiency of the biological carbon pump. Biogeosciences. 2017; 14(1): 177-186."

**Line 428-429** in the revised MS: "5. Zhang GH, Cao WY, Chen YY. Effects of lake stocking fishery on lake ecosystem in China. Acta Hydrobiol Sinica. 1997; 21(3): 271-280."

4. **Line 65-66** “..., indicating that the water quality improved after the removal of the seine.”

Need to add more detail on aquaculture facilities in Yangtze River.

**Answer:**

Thank you for your careful suggestions. Aquaculture facilities are mostly seine, cage and so on, so we use seine in the manuscript to make a general name.

5. **Line 66-69** “Susong County’s water production dropped sharply from 90400 t in 2017 (http://www.susongbbs.com/1408365-1-1.html) to 83900 t in 2018 (http://www.ahmhxc.com/tongjigongbao/14909_2.html).”

As a reference use of URL is very weak citation method. Please try to find and mention author or editor name and year. Hope you can understand.

**Answer:**

Thank you for your careful suggestions. I got the data in this sentence from the local statistical annual report, and I couldn't find the relevant literature. I'm deeply sorry. And i deleted the links in the resubmitted manuscript.

Line 65-66 in the revised MS: " Susong County’s water production dropped sharply from 90400 t in 2017 to 83900 t in 2018."

6. **Line 72-78** “...of lake environmental factors, composition, density and species diversity of metazooplankton with the purpose of: ...”

Please carefully correct the sentence structures of objectives.

**Answer:**

Thank you for your careful suggestions. In revised version, we have rewrite the sentence as follows.

**Line 69-75** in the revised MS: "From the summer of 2018 to the spring of 2019, we monitored the restoration of aquatic plants, parameters of lake environmental factors, composition, density and species diversity of metazooplankton. The aims of this study are as follows: (1) analyze the impact of aquatic plants recovering rapidly after the removal of enclosure aquaculture on water environment and metazooplankton; and (2) evaluate the main ecological factors influencing metazooplankton. The study is expected to provide a scientific basis for the restoration and management of lake water ecosystems in the Yangtze River basin."

**Material and method**

7. **Line 89** “... a total of 26 sample points (Fig 1, including seven sites in Lake Bo, nine sites in ...”

Sampling points

Please specify the sampling seasons such as summer, monsoon, winter..

**Answer:**

Thank you for your careful suggestions. In revised version, we have made a supplement as follows.

**Line 88-92** in the revised MS: "... a total of 26 sample points ...once per seasonfrom August 2018 to April 2019 (August 2018 is summer, November 2018 is autumn, January 2019 is winter, and April 2019 is spring). ”

8. **Line 93** “Metazooplankton is mainly composed of rotifera, cladocera and copepoda. We collected...”

Repeated sentence

**Answer:**

Thank you for your careful suggestions. I deleted the repeated sentence.

**Line 94** in the revised MS: "We collected... ”

9. **Line 99-100** “The collection method of qualitative samples of cladocera and copepoda is the same as that of rotifera qualitative samples. ”

Please try to combine the collection methods of Rotifera, Cladocera and Copepoda with specific mesh size of plankton net. Description of fixation methods could be differed from each other.

**Answer:**

Thank you for your careful suggestions. In revised version, we have made a supplement as follows.

**Line 99-101** in the revised MS: "The collection method of qualitative samples of cladocera and copepoda was the same as that of rotifera qualitative samples, but the water samples were filtered using a No.13 plankton net and fixed with 4% formaldehyde solution. ”

10. **Line 107-115** “At each sampling site, we collected an appropriate amount of water samples while taking metazooplankton samples. We... ”

Try to describe more specifically with related references and instrument models and number.

**Answer:**

Thank you for your careful suggestions. In revised version, we have made a supplement as follows.

**Line 108-116** in the revised MS: "At each sampling site, we collected an appropriate amount of water samples while taking metazooplankton samples. We stored the water samples at low temperature and brought them back to the laboratory according to the description of SEPB [22] for the measure of total nitrogen (TN), total phosphorus (TP), ammonium nitrogen (AN), nitrate nitrogen (NN) and other indicators. Using the 90% acetone extract+spectrophotometry measurement, we analyzed the Chlorophyll.α (Chl α) concentration, and then measured the turbidity (Turb; Hach, America) with the turbidimeter. We conducted an on-site measurement of water depth (WD), water temperature (WT), transparency (SD; Secchi disk), dissolve oxygen (DO), conductivity (Cond) and other water quality parameters.”

**Line 475-477** was the matching literature in the revised MS: "22. State Environmental Protection Bureau (SEPB). Methods of Monitoring and Analysis for Water and Wastewater, fourth ed. China Environmental Science Press, Beijing (in Chinese); 2002."

11. **Line 116** “2.3 Data analyses ..... ”

Data analysis methods are looks fine. I appreciate the description.

**Answer:**

Thank you for your recognition. We'll keep trying.

**Result**

12. **Line 140** “3. Result ..... ”

Results section is also looks fine. Well described in detail. I have on critical comment on it. Results are meets the objectives. I appreciate the efforts of the authors on it.

**Answer:**

Thank you for your recognition and detailed suggestions. We will answer your questions one by one.

13. **Line 166-167** “At the end of 2017, the aquaculture seine of the Huayanghe Lakes was removed, and the aquatic plants quickly recovered. ”

Please mention the references

**Answer:**

Thank you for your detailed suggestions. However, this result was observed in the field during our own field sampling, so we could not provide literature. Please forgive me.

**Discussion**

14. **Line 239** “4. Discussion ”

Very sorry to say, I am not convinced with the discussion. You have very good results. If you try harder, I believe you can write more attractive discussion. I request you to take my comments positively. I appreciate this research very much.

**Answer:**

Thank you for your kind suggestions. I will answer your comments one by one. Forgive me for my limitations.

15. **Line 240** “4.1 Effect of the removal of enclosure aquaculture on aquatic plants and water environment .... ”

I believe this research article will be a scientific resource. If you just modify your discussion with more references. Please try to make it readable with professional English.

**Answer:**

Thank you for your kind suggestions. We will answer your questions one by one.

16. All parts of discussion need to make to convenient to the readers. Need to add more references and discuss very constructively. Please make it readable and scientific.

**Answer:**

Thank you for your kind suggestions. We added new references and present it in the corresponding answers.

17. **Line 251-253** “Yang and Li [30] reported in the high-density seine fish culture test area in Lake East Taihu that aquatic plants were degraded due to fish culture pollution.”

Please reform this term "fish culture pollution" or try to define and describe more conveniently.

**Answer:**

Thank you for your careful suggestions. In revised version, we rewrote it as follows.

**Line 252-254** in the revised MS: "High-density net-enclosed fish culture affected the surrounding environment and aquatic plants due to its pollution effect and physical barrier effect, causing vegetation degradation[30]. ”

18. **Line 257-260** “Aquatic plants and phytoplankton together constitute the primary producer in the lake ecosystem, which are the starting point of the food chain and play an important role in the material cycle and energy flow. Therefore, aquatic plants have an important influence on the structure and function of the lake ecosystem.”

**Answer:**

Thank you for your careful suggestions. In revised version, we add the literature as follows.

**Line 256-262** in the revised MS: "Aquatic plants and phytoplankton ... Therefore, aquatic plants ... the lake ecosystem [31-32]. ”

**Line 503-505** was the matching literature in the revised MS: "31. Effiong KS, Inyang AI, Robert UU. Spatial distribution and diversity of phytoplankton community in Eastern Obolo River Estuary, Niger Delta. academicjournals. 2018; 9(1):1-14."

**Line 506-507** was the matching literature in the revised MS: "32. Xun Y. Application of macrophytes in water pollution control. Leather Manu Environ Technol. 2020; 1(01): 92-94."

19. **Line 261-264** “Analyzing the results of the water quality survey after the removal of purse seine in the Huayanghe Lakes, it was found that, compared with the previously published data by Xu (2016) and Zhang et al. (2019), the TN and TP contents were significantly reduced in summer, but the overall trend was increasing.”

Sentence structures are very weak so difficult to understand the information.

**Answer:**

Thank you for your careful suggestions. In revised version, we rewrote it as follows.

**Line 263-265** in the revised MS: "Compared with previous results [6,33], the results of this study showed that the contents of TN and TP decreased significantly in summer when aquatic plants grew vigorously, but showed an upward trend throughout the year.”

20. **Line 264-267** “Xue et al. (2010) found that, after the removal of the aquaculture net in Lake Dianshan, the concentration of TN and TP in Kunshan water increased in general, SD decreased, and DO also increased to a certain extent.”

Please define aquaculture net. It's better if you revise the references again and write more constructively.

**Answer:**

Thank you for your careful suggestions. In revised version, we rewrote it as follows.

**Line 265-267** in the revised MS: "Xue et al. [34] studied the Lake Dianshan after the demolition of the aquaculture facilities, and also found that the concentration of TN and TP in the water increased overall.”

21. **Line 270-271** “However, in the process of seasonal decay, large aquatic plants will also release their own nutrients into the water body, leading to the increase of nutrients.”

Please describe seasonal decay.

**Answer:**

Thank you for your careful suggestions. In revised version, we rewrote it and added the literature as follows.

**Line 270-273** in the revised MS: "However, in the process of natural succession and seasonal changes, the growth decline, death and litter decomposition of large aquatic plants are necessary stages; and the decomposition of their residues and the release of nutrients lead to the increase of nutrients [37-38]. ”

**Line 520-521** was the matching literature in the revised MS: "37. Zhang LJ, Ye C, Li CH, Song XF. The effect of submerged macrophytes decomposition on water quality. Res Environ Sci. 2013; 26(2): 145-151."

**Line 522-523** was the matching literature in the revised MS: "38. Wang Q, Huang W, Wu T, Hao ZX. Research on decomposition process of macrophyte. J Green Sci Technol. 2020; (18): 1-3."

22. **Line 287-290** “They are widely used to treat eutrophication water bodies to reduce nitrogen and phosphorus levels, improve water transparency, and gradually restore other aquatic plants and the whole ecological environment.”

References ?

**Answer:**

Thank you for your careful suggestions. In revised version, we added the literature as follows.

**Line 289-291** in the revised MS: "They are widely used to treat eutrophication ... ecological environment [41-42]. ”

**Line 534-535** was the matching literature in the revised MS: "42. Yan BM, Li XJ. Application of Aquatic Plants in River and Lake Water Treatment. Northwest Hydropower. 2020; (S2): 22-25."

23. **Line 291-292** “4.2 Effects of aquatic plants restoration on metazooplankton after the removal of enclosure aquaculture”

It's highlighted here

**Line 318-320** “Hu's (1999) research on metazooplankton in five lakes, including Baoan Lake in Hubei in the middle and lower reaches of the Yangtze River, discovered that metazooplankton species and density were closely related to aquatic vegetation.”

**Line 327-330** “Zeng Lei et al. (2018) reported in their study that species richness of large zooplankton were obviously enhanced in the restoration process of aquatic plants. It has been also found that the proportion of metazooplankton was highest in cladocera and copepoda in Lake Bo.”

**Answer:**

Thank you for your careful suggestions. In revised version, we have rewritten some sentences as follows.

**Line 317-319** in the revised MS: "Hu et al. [51] showed that in lakes with different aquatic plants coverage, the species diversity and density of metazooplankton would increase with the rise of aquatic plants coverage."

**Line 326-330** in the revised MS: "This is consistent with the results of other scholars[8, 12], and the proportion of cladocera and copepoda in the abundance of metazooplankton in Lake Bo is higher. That is, the species richness of macrozooplankton is significantly enhanced during the restoration of aquatic plants."
